# Supplementary material for: What matters most? A qualitative study exploring priorities for supportive interventions for people with tuberculosis in urban Viet Nam
Source: BMJ Open. 2023 Aug 23;13(8):e076076. doi: 10.1136/bmjopen-2023-076076 (PMC10450053; doi:10.1136/bmjopen-2023-076076)
Supplement: Supplementary data [file bmjopen-2023-076076supp001.pdf]

Appendix A: Details of data collection

Participant group	Number of participants	Type	Location	Duration (minutes)
People with successfully treated DS-TB	5¶	FGD	Ha Noi (NGO office)	146
People with unsuccessfully treated DS-TB	4	FGD	Ha Noi (NGO office)	103
District TB Unit HCP	5	FGD	Ha Noi (NGO office)	91
People with successfully treated DS-TB	3	FGD	Ho Chi Minh City (NGO office)	138
People with unsuccessfully treated DS-TB	2	FGD	Ho Chi Minh City (NGO office)	113
People with MDR-TB	4	FGD	Ha Noi (Ha Noi Lung Hospital)	109
People with MDR-TB	6	FGD	Ho Chi Minh City (NGO office)	188
National/Provincial TB HCP	4*	KII	Ha Noi (Zoom)	46-90

¶ Indicates that at least one participant was accompanied by their caregiver
**Four separate KII were conducted.*

Smith I, et al. BMJ Open 2024; 13:e076076. doi: 10.1136/bmjopen-2023-076076
