# Supplementary material for: What matters most? A qualitative study exploring priorities for supportive interventions for people with tuberculosis in urban Viet Nam
Source: BMJ Open. 2023 Aug 23;13(8):e076076. doi: 10.1136/bmjopen-2023-076076 (PMC10450053; doi:10.1136/bmjopen-2023-076076)
Supplement: Supplementary data [file bmjopen-2023-076076supp002.pdf]

Appendix B: Topic guides for data collection

Focus Group Discussions (DTU and Provincial TB hospital staff)

Prior experiences of providing TB treatment support

- What existing social support resources are available for the TB patients that you treat?
    - How do you link your patients to existing resources?

Barriers to TB treatment

- From your experience, what is the biggest obstacle that prevents patients from completing treatment?
- What are the main factors that cause catastrophic costs or poverty during TB treatment?

Types of social support

- If you could design any intervention to support the TB patients you treat, what would be provided?

Social health insurance

- Which types of TB patients should receive health insurance? Why?
- Who should provide SHI to TB patients?
- How effective is SHI at alleviating the costs of TB care?

TB Education

- On what types of information should TB patients receive counselling and education?
- When should this information be given during the duration of treatment?
- If you were given any teaching aids to improve the patient counselling, what would those teaching aids look like?

Nutritional support

- What types of food are needed by TB patients?
- What types of supplements are needed during TB treatment?
- Which types of patients should be offered nutritional support, if it is offered?
- For how long do TB patients need nutritional support?
    - Prompt: during the intensive phase or throughout all of treatment?
- What are the benefits of providing food and/or supplements?
- Do you see any drawbacks to the provision of food and/or supplements?

Cash transfers

- Which types of TB patients should receive the cash transfer? Why?
- For how long should cash be given to TB patients?
- What are the benefits and drawbacks of cash transfers compared to other types of social support?
- How should cash be spent?
- How should cash not be spent?

Loans

- What was your need for credit during TB care?
- How do you feel about providing loans to TB patients? Whose role should it be to provide loans?
- How much credit should be given?
- How should TB patients spend the loan money?
- How would loans address the needs of TB patients?
- What should the payment plan consist of?
- How should we collect on the loan?

Transport vouchers

- How do you feel about transportation vouchers being given to TB patients?
- How do transport vouchers address the needs of TB patients?
- Which types of patients should be offered transport vouchers, if they are offered?
- How willing do you feel TB patients would be to participate in a project that provided transport vouchers?
- What challenges do you foresee in providing transport vouchers to TB patients?

Patient support groups

- What is your opinion of TB patient support groups?
- Which types of TB patients would benefit the most from patient support groups?
- What types of benefits would TB patients gain from a TB support group?
- How willing do you feel TB patients would be to participate patient support groups?
- During which stage of TB treatment would a patient support group be most effective to support the patient?
- Who is the most effective type of person to lead the patient support group?

    - Prompt: Peer-led, DTU-led or psychologist-led
- How would you feel about hosting a patient support group?

Access to a psychologist/ psychiatrist for DS-TB patients

- What types of psychological or psychiatric issues do your DS-TB patients face?
- What mental health support services do DS-TB patients require?
- What resources are currently available to them for psychological or psychiatric problems?
- How willing do you feel DS-TB patients would be to meet with a mental health professional during their treatment?
- How would better access to a mental health professional improve DS-TB treatment outcomes?

Priority access to testing and preventative therapy

- How can patient's families best be supported to go for TB testing?
- What barriers do families of people with TB face in accessing TB testing?
- What is the best way to improve LTBI treatment initiation rates among TB contacts?
- What barriers do families of people with TB face in accessing LTBI testing and treatment?

Prioritization to improve treatment outcomes

- Following this discussion, what do you feel is the most important type of support for TB patients to improve their treatment outcomes? Why?
- What are the second and third most important types of support for TB treatment?

Prioritization to reduce catastrophic costs

- What do you feel is the most important type of support for TB patients to reduce catastrophic costs? Why?
- What are the second and third most important types of support to reduce patient costs?

Is there anything else you would like to tell us?

Focus Group Discussions (Current/Former TB patients)

Experiences on TB treatment

- When you were on TB treatment, what types of challenges did you face?
- What types of support did you need? Why?

Barriers to TB treatment

- From your experience, what is the biggest obstacle that prevents patients from completing treatment?
- What are the main factors that cause catastrophic costs or poverty during TB treatment?

Types of support

- If you could design any type of program to help TB patients, not thinking about the cost, what would that program include?

Social health insurance

- Which types of TB patients should receive health insurance? Why?
- Who should provide SHI to TB patients?
- How effective is SHI at alleviating the costs of TB care?

Education

- On what types of information should TB patients receive counselling?
- When should this information be given during the duration of treatment?
- Thinking back the to the counselling you received, if you could change anything about this counselling, what would you change?

Nutritional support

- What types of food are needed by TB patients?
- What types of supplements are needed during TB treatment?
- Which types of patients should be offered nutritional support, if it is offered?
- For how long do TB patients need nutritional support?
    - Prompt: during the intensive phase or throughout all of treatment?
- What are the benefits of providing food and/or supplements?
- Do you see any drawbacks to the provision of food and/or supplements?

Cash transfers

- Which types of TB patients should receive the cash transfer? Why?
- For how long should cash be given to TB patients?
- What are the benefits and drawbacks of cash transfers compared to other types of social support?

- How should cash be spent?
- How should cash not be spent?

<u>Vocational training</u>

- How helpful do you feel vocational training would be to TB patients?
    - Prompt: During treatment vs. after treatment
    - Prompt: short-term vs. long-term benefits
- Which types of patients should be offered vocational training, if it is offered?
- How willing do you feel TB patients would be to participate?
- What would be the benefits of vocational training to TB patients?
- Which barriers would TB patients face completing the vocational training?

<u>Transport vouchers</u>

- How do you feel about transportation vouchers being given to TB patients?
- How do transport vouchers address the needs of TB patients?
- Which types of patients should be offered transport vouchers, if they are offered?
- How willing do you feel TB patients would be to participate in a project that provided transport vouchers?
- What challenges do you foresee in providing transport vouchers to TB patients?
- What are the benefits of transport vouchers compared to cash payments?

<u>Patient support groups</u>

- What is your opinion of TB patient support groups?
- What types of challenges do patient support groups best address?
- What issues should patient support groups discuss?
- Which types of TB patients would benefit the most from patient support groups?
- What types of benefits would TB patients gain from a TB support group?
- How willing do you feel TB patients would be to participate patient support groups?
- During which stage of TB treatment would a patient support group be most effective to support the patient?
- Who is the most effective type of person to lead the patient support group?
    - Prompt: Peer-led, DTU-led or psychologist-led
- How would you feel about hosting a patient support group?

<u>Access to a psychologist/ psychiatrist for DS-TB patients</u>

- What mental health support services did you require during your treatment?
- What resources were available to you for psychological or psychiatric problems, if any?
- How willing do you feel DS-TB patients would be to meet with a mental health professional during their treatment?
- How would access to a mental health professional improve DS-TB treatment outcomes?

Access to testing and preventative therapy

- How can patient's families best be supported to go for TB testing?
- What barriers do families of people with TB face in accessing TB testing?
- What is the best way to improve LTBI treatment initiation rates among TB contacts?
- What barriers do families of people with TB face in accessing LTBI testing and treatment?

Prioritization to improve treatment outcomes

- Following this discussion, what do you feel is the most important type of support for TB patients to improve their treatment outcomes? Why?
- What are the second and third most important types of support for TB treatment?

Prioritization to reduce catastrophic costs

- What do you feel is the most important type of support for TB patients to reduce catastrophic costs? Why?
- What are the second and third most important types of support to reduce patient costs?

Is there anything else you would like to tell us?

KIIs (National and Provincial TB staff)

Barriers

- From your experience, what is the biggest obstacle that prevents patients from completing treatment?
- What are the main factors that cause catastrophic costs or poverty during DR-TB treatment?

Prior experiences of providing treatment support to people with DR-TB

- What existing support resources are available for the DR-TB patients that you treat?
  - How do you link your patients to existing resources?
  - How effective do you find these sources to be?
    - Describe the gaps in this support
    - How adequate do you find this support to be?
    - How easy or difficult is it for people with DR-TB to obtain economic or psychological support?
- Which groups of people with DR-TB require economic and psychological support?

Intervention design

- If you could design any intervention to support DR-TB patients, what would be provided?
  - Why would this be provided?
  - Who should receive it?
    - Differentiate packages of support based on levels of disability/ poverty status/ income/ number of prior episodes of TB?
  - For how long should this support be provided?
  - What should be required of the DR-TB patient to receive the support?
  - What should *not* be provided?
  - How can technology be used to support this intervention?
    - Prompt for remote monitoring and drug delivery through shipper apps

Priortization to improve treatment outcomes & catastrophic costs

- Even if this differs from the intervention you proposed, what do you feel is the most important type of support for DR-TB patients to improve DR-TB *treatment outcomes*? Why?
- What do you feel is the most important type of support for DR-TB patients to reduce *catastrophic costs*? Why?

Provision of support

- In your role, do you have capacity to assist with the provision of support to people with DR-TB?
- If this work was to be conducted in the facility where you work, who should provide it?

Is there anything else you would like to tell us?

<u>Focus Group Discussions (Current/Former DR-TB patients)</u>

Experiences on TB treatment

- While on treatment, what types of challenges have you faced?
- What types of support do you need during DR-TB treatment? Why?

Barriers to TB treatment

- From your experience, what is the biggest obstacle that prevents patients from completing treatment?
- What are the main factors that cause catastrophic costs or poverty during DR-TB treatment?

Types of support

- If you could design any type of program to help people through DR-TB treatment, not thinking about the cost, what would that program include?

***If the participants mention a type of support listed below, then ask the questions for that form of support. If they do not bring up a form of support that is outlined below, then prompt them to discuss.*

<u>Social health insurance</u>

- Which types of DR-TB patients should receive health insurance? Why?
- Who should provide SHI to DR-TB patients?
- For how long should SHI be provided to people with DR-TB?
- How effective is SHI at alleviating the costs of DR-TB care?

<u>Education</u>

- On what types of information should DR-TB patients receive counselling?
- When should this information be given during the duration of treatment?
- Thinking back the to the counselling you received, if you could change anything about this counselling, what would you change?

<u>Nutritional support</u>

- What types of food are needed for people with DR-TB?
- What types of supplements are needed during DR-TB treatment?
- Which types of people with DR-TB should be offered nutritional support, if it is offered?

  - Differentiate packages of support based on levels of disability/ poverty status/ income/ number of prior episodes of TB?
- For how long do DR-TB patients need nutritional support?
    - Prompt: during the intensive phase or throughout all of treatment?
- What are the benefits of providing food and/or supplements?
- Do you see any drawbacks to the provision of food and/or supplements?

Cash transfers

- Which types of DR-TB patients should receive the cash transfer? Why?
  - Differentiate cash amounts based on levels of disability/ poverty status/ income/ number of prior episodes of TB?
- For how long should cash be given to people with DR-TB?
- What are the benefits and drawbacks of cash transfers compared to other types of social support?
- How should cash be spent?
- How should cash not be spent?

Transport vouchers

- How do you feel about transportation vouchers being given to people with DR-TB?
- How do transport vouchers address the needs of people with DR-TB?
- Which types of patients should be offered transport vouchers, if they are offered?
  - Differentiate amount of transport voucher based on distance from clinic/ levels of disability/ poverty status/ income/ number of prior episodes of TB?
- How willing do you feel DR-TB patients would be to participate in a project that provided transport vouchers?
- For how long should transport vouchers be given to people with DR-TB?
- What are the benefits of transport vouchers compared to cash payments?

At-home medicine delivery

- How do you feel about medicine being delivered to the homes of people with DR-TB through an app such as Grab, Ahamove, Bee, or another shipper?
- How would medicine delivery address the needs of people with DR-TB?
- Which types of patients should be offered medicine delivery?
- How willing do you feel DR-TB patients would be to receive medicine delivery?
- For how long should medicine be delivered to the homes of people with DR-TB?

Remote monitoring

- How do you feel about attending appointments with your doctor online?
- How would online appointments address the needs of people with DR-TB?
- Which types of patients should be offered remote monitoring or telemedicine?
- How willing do you feel DR-TB patients would be to receive online consultations of in-person meetings?

Patient support groups

- What is your opinion of DR-TB patient support groups?
    - Prompt- in person vs. online support groups
- What types of challenges do patient support groups best address?
- What issues should patient support groups discuss?
- Which types of DR-TB patients would benefit the most from patient support groups?
- What types of benefits would DR-TB patients gain from a support group?
- How willing do you feel people with DR-TB would be to participate patient support groups?
    - Prompt- in person vs. online support groups
- During which stage of DR-TB treatment would a patient support group be most effective to support the patient?
- Who is the most effective type of person to lead the patient support group?
    - Prompt: Peer-led, DTU-led or psychologist-led
- How would you feel about hosting a patient support group?

Access to a psychologist/ psychiatrist for DR-TB patients

- What mental health support services have you required during your treatment?
- What resources were available to you for psychological or psychiatric problems, if any?
- How willing do you feel DR-TB patients would be to meet with a mental health professional during their treatment?
- How would access to a mental health professional improve DR-TB treatment outcomes?

Access to testing and preventative therapy

- How can patient’s families best be supported to go for TB testing?
- What barriers do families of people with TB face in accessing TB testing?
- What is the best way to improve LTBI treatment initiation rates among DR-TB contacts?

- What barriers do families of people with DR-TB face in accessing LTBI testing and treatment?

Priortization to improve treatment outcomes & catastrophic costs

- Following this discussion, what do you feel is the most important type of support for DR-TB patients to improve their *treatment outcomes*? Why?
- What do you feel is the most important type of support for TB patients to reduce catastrophic costs? Why?

Is there anything else you would like to tell us?
