# Supplementary material for: What matters most? A qualitative study exploring priorities for supportive interventions for people with tuberculosis in urban Viet Nam
Source: BMJ Open. 2023 Aug 23;13(8):e076076. doi: 10.1136/bmjopen-2023-076076 (PMC10450053; doi:10.1136/bmjopen-2023-076076)
Supplement: Author's manuscript [file bmjopen-2023-076076.draft_revisions.pdf]

BMJ Open is committed to open peer review. As part of this commitment we make the peer review history of every article we publish publicly available.

When an article is published we post the peer reviewers' comments and the authors' responses online. We also post the versions of the paper that were used during peer review. These are the versions that the peer review comments apply to.

The versions of the paper that follow are the versions that were submitted during the peer review process. They are not the versions of record or the final published versions. They should not be cited or distributed as the published version of this manuscript.

BMJ Open is an open access journal and the full, final, typeset and author-corrected version of record of the manuscript is available on our site with no access controls, subscription charges or pay-per-view fees (http://bmjopen.bmj.com).

If you have any questions on BMJ Open's open peer review process please email info.bmjopen@bmj.com

BMJ Open

What matters most? A qualitative study exploring priorities for supportive interventions for people with TB in urban Viet Nam

Journal:	*BMJ Open*
Manuscript ID	bmjopen-2023-076076
Article Type:	Original research
Date Submitted by the Author:	29-May-2023
Complete List of Authors:	Smith, Isabel; Karolinska Institute, WHO Collaborating Centre for Social Medicine and Tuberculosis, Department of Global Public Health Sciences<br>Forse, Rachel; Karolinska Institutet, WHO Collaborating Centre for Social Medicine and Tuberculosis, Department of Global Public Health Sciences; Friends for International TB Relief, TB Programs<br>Sidney, Kristi; Karolinska Institutet, WHO Collaborating Centre for Social Medicine and Tuberculosis, Department of Global Public Health Sciences<br>Thanh, Nguyễn Thị; Centre for Development of Community Health Initiatives<br>Nguyen, Lan; IRD VN<br>Phan, Thi Hoang Yen; Centre for Development of Community Health Initiatives<br>Nguyen, Han; Friends for International Tuberculosis Relief<br>Codlin, Andrew; Friends for International Tuberculosis Relief; Karolinska Institute, WHO Collaborating Centre for Social Medicine and Tuberculosis, Department of Global Public Health Sciences<br>Vo, Luan ; Friends for International TB Relief,<br>Nguyen, Nga Thi Thuy; Friends for International Tuberculosis Relief<br>Khan, Amera; Stop TB Partnership<br>Creswell, Jacob; Stop TB Partnership,<br>Pham Huy, Minh; USAID Vietnam<br>Basu, Lopa; USAID Vietnam<br>Lönnroth, Knut; Karolinska Institute, WHO Collaborating Centre for Social Medicine and Tuberculosis, Department of Global Public Health Sciences<br>Nguyen, Binh Hoa; Vietnam National Tuberculosis Program<br>Nguyen, Viet Nhung; National Lung Hospital , National TB Program<br>Atkins, Salla; Tampere University, New Social Research and Faculty of Social Sciences; Karolinska Institute, WHO Collaborating Centre for Social Medicine and Tuberculosis, Department of Global Public Health Sciences
Keywords:	Tuberculosis < INFECTIOUS DISEASES, Health policy < HEALTH SERVICES ADMINISTRATION & MANAGEMENT, Public health < INFECTIOUS DISEASES

For peer review only - http://bmjopen.bmj.com/site/about/guidelines.xhtml

I, the Submitting Author has the right to grant and does grant on behalf of all authors of the Work (as defined in the below author licence), an exclusive licence and/or a non-exclusive licence for contributions from authors who are: i) UK Crown employees; ii) where BMJ has agreed a CC-BY licence shall apply, and/or iii) in accordance with the terms applicable for US Federal Government officers or employees acting as part of their official duties; on a worldwide, perpetual, irrevocable, royalty-free basis to BMJ Publishing Group Ltd ("BMJ") its licensees and where the relevant Journal is co-owned by BMJ to the co-owners of the Journal, to publish the Work in this journal and any other BMJ products and to exploit all rights, as set out in our licence.

The Submitting Author accepts and understands that any supply made under these terms is made by BMJ to the Submitting Author unless you are acting as an employee on behalf of your employer or a postgraduate student of an affiliated institution which is paying any applicable article publishing charge ("APC") for Open Access articles. Where the Submitting Author wishes to make the Work available on an Open Access basis (and intends to pay the relevant APC), the terms of reuse of such Open Access shall be governed by a Creative Commons licence – details of these licences and which Creative Commons licence will apply to this Work are set out in our licence referred to above.

Other than as permitted in any relevant BMJ Author's Self Archiving Policies, I confirm this Work has not been accepted for publication elsewhere, is not being considered for publication elsewhere and does not duplicate material already published. I confirm all authors consent to publication of this Work and authorise the granting of this licence.

For peer review only

What matters most? A qualitative study exploring priorities for supportive interventions for people with TB in urban Viet Nam

Authors

Isabel Smith[1], Rachel Forse[1,2], Kristi Sidney-Annerstedt[1], Thanh Thi Nguyen[3], Lan Nguyen[4], Thi Hoang Yen Phan[3], Han Nguyen[2], Andrew J. Codlin[1,2], Luan Nguyen Quang Vo[2], Nga Nguyen[2], Amera Khan[5], Jacob Creswell[5], Minh Pham[6], Lopa Basu[6], Knut Lönnroth[1], Hoa Binh Nguyen[7], Nhung Viet Nguyen[7], Salla Atkins[1,8]

1. WHO Collaborating Centre for Social Medicine and Tuberculosis, Department of Global Public Health, Karolinska Institute, Stockholm, Sweden
2. Friends for International TB Relief (FIT), 1/21 Le Van Luong, Nhan Chinh, Thanh, Xuan, Ha Noi, Viet Nam
3. Centre for Development of Community Health Initiatives (CHI), 1/21 Le Van Luong, Nhan Chinh, Thanh Xuan, Ha Noi, Viet Nam
4. IRD VN, 68B Nguyen Van Troi, Ward 3, Phu Nhuan, Ho Chi Minh City, Viet Nam
5. Stop TB Partnership, Chem. du Pommier 40, 1218 Le Grand-Saconnex, Switzerland
6. United States Agency for International Development (USAID), 15/F, Tung Shing Building, 2 Ngo Quyen, Ha Noi, Viet Nam
7. National Lung Hospital/National TB Control Programme, 463 Hoang Hoa Tham, Vinh Phu, Ba Dinh, Ha Noi, Viet Nam
8. Faculty of Social Sciences, Tampere University, Arvo Ylpön katu 34, Tampere, Finland

Corresponding author

Rachel Forse

rachel.forse@tbhelp.org

Abstract

Introduction: The health and economic burden of Tuberculosis (TB) in urban Viet Nam is high. Social protection and support interventions can improve treatment outcomes and reduce costs. However, evidence regarding optimal strategies in this context is lacking. This study aimed to increase understanding of what people with TB and TB healthcare providers (HCPs) perceive as important to improve TB treatment outcomes and reduce costs.

Methods: We conducted qualitative focus group discussions (7 groups, n=30) and key informant interviews (n=4) with people with drug-susceptible and multidrug-resistant TB and HCPs in Ha Noi and Ho Chi Minh City. Topic guides covered perspectives on and prioritization of different forms of social protection and support. Data were analysed using reflexive thematic analysis and interpreted using a Framework for Transformative Social Protection.

Results: We identified three themes and seven sub-themes. The first theme, 'Existing financial safety nets are essential, but could go further to support people affected by TB', highlights that support to meet the medical costs of TB treatment and flexible cash transfers are a priority for people with TB and HCPs. The second, 'It is important to promote "physical and spiritual health" during TB treatment', demonstrates that extended psychosocial and nutritional support would encourage people with TB during their treatment. The third, 'Accessibility and acceptability are critical in designing social support interventions for

For peer review only - http://bmjopen.bmj.com/site/about/guidelines.xhtml

people with TB', shows the importance of ensuring that support is easily accessible and proportional to the needs of people with TB and their families.

Conclusions: Interventions that extend beyond the medical sector are required to address the needs of people affected by TB in Viet Nam, including social protection and social support measures. This study can inform the design of stronger person-centred interventions to advance progress towards the goals of the World Health Organization's End TB Strategy.

Key messages (3-5 sentences).

- **What is already known on this topic?** People with TB in Viet Nam face catastrophic costs during their TB treatment course, which perpetuates a cycle of poverty and disease, often leading to long-term financial, physical and mental health impacts. Supportive interventions can relieve these costs and improve treatment outcomes; however, not enough is known about which specific types of interventions are important to people affected by TB and healthcare providers in this specific context.
- **What this study adds.** Our findings highlight that both people with TB and TB healthcare providers in urban Viet Nam identify a need for a broader package of support services that combines financial risk protection, psychosocial support and support for the management of side effects.
- **How this study might affect research, practice or policy.** This study can inform the types of support that government agencies and other actors choose to provide for people with TB and adds to a growing body of evidence on routes to achieving the goals of the End TB Strategy. In broader terms this study emphasises how social support interventions can improve the lives of people affected by TB and promote full completion of the TB treatment course, thereby contributing to reduced transmission, morbidity and mortality.

For peer review only

Introduction

Tuberculosis (TB) caused 1.6 million deaths in 2021 (1). TB disproportionately affects poorer populations who are more vulnerable to TB infection, development of active TB disease, and worse treatment outcomes (1–4). Further, TB can push affected households into poverty (5,6) through the accrual of direct and indirect costs. These can amount to catastrophic costs, defined by the WHO as total costs due to TB exceeding 20% of annual household income (1). The impoverishing effects of TB may have been compounded by COVID-19, particularly for more vulnerable populations (7). Eliminating TB will require both biomedical innovation and socio-economic interventions, as outlined in the World Health Organization's (WHO) End TB Strategy (8).

The provision of social protection schemes must be a part of the response to end TB. There is consistent evidence that spending on broad social protection, including population-level poverty reduction schemes such as pensions and child support, is inversely associated with TB incidence and mortality (9). Some estimates indicate that expansion of social protection initiatives could reduce global TB incidence by up to 76% (10). Furthermore, social protection schemes for people with TB such as cash transfers and transport vouchers have been shown to defray out-of-pocket costs and improve treatment outcomes in various high-burden settings (11, 12). While most social protection interventions for TB-affected household focus on financial support (13), social and psychological support interventions have also contributed to improved treatment outcomes and reduced costs (14).

Viet Nam is one of the world's highest burden countries for TB and multidrug-resistant TB (MDR-TB) (15), and TB incidence in Viet Nam is particularly high in urban areas (16). Despite the provision of free diagnostics and anti-TB treatment by the National TB Program (NTP) and high coverage of social health insurance (SHI) covering medical costs (17), a 2016 patient cost survey found that 63% of households containing a person with drug-susceptible (DS-TB) and 98% of households containing a person with MDR-TB experienced catastrophic costs due to the disease (18).

In response, Viet Nam's NTP identified the impoverishing effects of TB as a priority for action (19) and established a national fund (Patients Support to Fight TB) to support poor and uninsured people with TB (for example, by purchasing SHI on their behalf) (18,19). However, there is limited context-specific understanding of the optimal ways in which supportive interventions can improve treatment outcomes and reduce costs for people with TB in urban Viet Nam. A prior study exploring the acceptability of cash transfers and SHI among people with TB, healthcare providers (HCPs), policymakers and members of the public in Viet Nam (Forse, Nguyen, Dam et al. 2023) indicated that further research into preferences for supportive interventions was required. Therefore, this study aims to increase understanding of what people with TB and HCPs in urban Viet Nam perceive as important in the design of interventions to reduce costs and improve TB treatment outcomes.

Methods

Study design

We conducted an exploratory qualitative study using semi-structured focus group discussions (FGDs) and key informant interviews to understand the perceptions of people with TB and TB HCPs in urban Viet Nam regarding supportive interventions to improve

For peer review only - http://bmjopen.bmj.com/site/about/guidelines.xhtml

treatment outcomes and reduce costs. The Consolidated Criteria for Reporting Qualitative Research checklist (20) was used to ensure comprehensive reporting.

Study setting

The study was conducted in two cities in Viet Nam, Ha Noi and Ho Chi Minh City, where a consortium of local NGO partners works with the NTP to deliver enhanced TB care and conduct operational research (21,22). Data were collected in July-September 2020 and February 2022 in the context of COVID-19.

The NTP provides anti-TB drugs and consultations free-of-charge in the public sector. At the time of the study, SHI could not be used for TB medications (18) but covered 80% of reimbursable medical expenditure for most people (23). To obtain SHI, people must register and pay an annual fee for the entire household (24). For most people, SHI is registered at the commune or district level and is not valid at provincial or national hospitals without a referral (25). People with TB meet other costs of treatment through out-of-pocket payments and social protection schemes supported by government programs, the Global Fund and other funding sources (18).

In 2021, the TB incidence rate in Viet Nam was 173 cases per 100,000 people and there were over 2,600 new cases of MDR-TB (1). Among those treated, treatment was successful in 91% of new and relapse cases and in 74% of MDR-TB cases (1).

Participant selection

Purposive sampling was used to select participants with a range of experiences of TB treatment who had not previously engaged in the NGOs' services. TTN contacted District TB Units (DTUs) in Ha Noi and Ho Chi Minh City who provided lists of potential participants: people who had been successfully or unsuccessfully[1] treated for bacteriologically-confirmed drug-susceptible (DS-) TB, people being treated for bacteriologically-confirmed MDR-TB with a 9- or 20-month regimen, and TB HCPs employed at national, provincial and district levels.

An assessment of information power (26) concluded that a moderate sample size was required to address the study's aim. The sample size was guided by estimates based on previous studies and data saturation was discussed following each FGD or interview. A total of 66 individuals were invited, but 33 (13 men, 20 women) declined to participate, mostly due to a lack of interest or distance. One participant was accompanied by their caregiver, who also gave consent to participate. Demographic characteristics are reported in Table 1.

Table 1: Participant demographic characteristics.

	Sex (n)		Total (n)
	**Female**	**Male**	
All Participants	12¶	21	33¶
Location			
Ha Noi	10¶	12	22¶

[1] Unsuccessfully treated defined as retreated or relapse cases.

For peer review only - http://bmjopen.bmj.com/site/about/guidelines.xhtml

For peer review only

Ho Chi Minh City	2	9	11
Participant Group			
People successfully treated for DS-TB	1¶	7	8¶
People unsuccessfully treated for DS-TB	0	6	6
People with MDR-TB	4	6	10
District TB HCPs	3	2	5
National/Provincial TB HCPs	3	1	4
Age Group			
18- 24 years	2	2	4
25 - 44 years	5	8	13
44 - 59 years	5	7	12
60 - 75 years	0¶	4	4¶

Note: ¶ Indicates that at least one participant was accompanied by their caregiver.

Data collection

Data were collected by female NGO staff trained and experienced in conducting qualitative research, and was directly overseen by TTN, a qualitative researcher and NGO project manager with five years of qualitative research experience. All participants were informed about the purpose of the study when they were invited to participate by telephone, although not provided with details about the interviewers' motivations or biases. They provided written informed consent prior to data collection. Participants were provided with refreshments and 300,000 VND ($12.73 USD, 23,571.8 VND: 1 USD, xe.com) to cover travel costs.

Four FGDs with people with DS-TB and one FGD with DTU HCPs were conducted in July-September 2020 in dedicated offices. Two FGDs with people with MDR-TB and four interviews with National/Provincial TB HCPs were conducted in February 2022 at the Ha Noi Lung Hospital, the NGO offices or over Zoom. FGDs were conducted in person and lasted 91-188 minutes. Key informant interviews were conducted via Zoom and lasted 46-90 minutes. The study aimed to recruit five participants per FGD, however some were smaller due to enrolment challenges and may be better classified as group interviews. No repeat interviews were conducted. Further details can be found in Appendix A.

Topic guides were developed iteratively by TTN, RF and KSA and included questions on challenges during TB treatment and different forms of support; complete topic guides are in Appendix B. Questions on digital support were added to later FGDs and interviews due to its increased use during the COVID-19 pandemic. Participants were also asked to rank interventions that they thought were most important for improving treatment outcomes and reducing costs.

FGDs were conducted in Vietnamese, audio recordings were transcribed by NGO staff and then translated into English by an external service provider in Viet Nam. The translations were checked by bilingual study staff. Field notes were recorded following the FGDs and KIIs. Participant data were pseudonymised, stored securely and not shared beyond the study team. Transcripts were not shared with participants for comment.

Patient and Public Involvement

For peer review only - http://bmjopen.bmj.com/site/about/guidelines.xhtml

People with TB, HCPs, policymakers and members of the public were involved in this research through the development of topic guides. Their responses to a prior qualitative interview study on the acceptability of cash and SHI (Forse, Nguyen, Dam et al. 2023) informed the development of a list of forms of social protection to explore further in this study. The results of this research were disseminated to government officials, the NTP and patient communities in roundtable discussions.

Data analysis

Data were analysed using reflexive thematic analysis (27, 28). IS, a global health masters student, developed the coding framework with SA, an experienced qualitative researcher, and coded the data using 'Dedoose' software (version 9.0.54) (29) before identifying themes based on recurrent patterns. The analysis was conducted through an iterative process. Codes, categories, themes and sub-themes were verified by SA, KSA and RF. An example is illustrated in Table 2.

Table 2: Examples of quotes with corresponding code descriptions, codes, themes and sub-themes.

Example quote	Code description	Code	Sub-theme	Theme
"There are many aspects that make people give up including travel expenses. So thanks to this voucher support during the treatment a lot of sick people would benefit."	The costs of TB are a barrier to completing treatment	Financial problems affect adherence	Enabling people with TB to meet medical costs is a priority for support	Existing financial safety nets are essential, but could go further to support people with TB
"When I was sad, my family encouraged me."	People with TB are encouraged and supported by friends and family	Emotional support from friends and family	Psychosocial support is fundamental for people with TB to "feel secure in treatment"	It is important to promote "physical and spiritual health" during TB treatment
"Besides, during the time of taking this medicine, it was very tiring and exhausting, so I had to drink a lot of water, take tonics during that course."	TB drugs can make people with TB feel tired and weak	Treatment makes you tired and weak	Nutritional supplementation is viewed as important for people with TB to be able to "fight against the disease"	It is important to promote "physical and spiritual health" during TB treatment
"When they know it, honestly, they are afraid to examine and test. If they are ignored about how it is dangerous, they shall still omit it."	Contacts of people with TB don't want to get tested	Contacts reluctant to test	Increasing access to TB diagnostic and preventive services is an important part of social protection	Accessibility and acceptability are critical in designing social support interventions for people with TB

Themes were interpreted in line with the Framework for Transformative Social Protection which defines social protection as a range of policies or initiatives which help vulnerable

For peer review only - http://bmjopen.bmj.com/site/about/guidelines.xhtml

people to move sustainably out of poverty by acting on their financial risk and enhancing their social status (30).

Results

We generated three themes and five sub-themes from the data, summarised in Table 3 and discussed below. Participants also spoke about challenges experienced during TB treatment which are covered extensively elsewhere (2,5,6,17,31). We have indicated where HCPs and people with TB differed in opinion, and used 'participants' they concurred.

Table 3: Themes and sub-themes identified during reflexive thematic analysis.

Themes		Sub-themes	
1	Existing financial safety nets are essential but could go further to support people with TB	1.1	Enabling people with TB to meet medical costs is a priority for support
		1.2	Flexible financial support can give people with TB more agency
2	It is important to promote "physical and spiritual health" during TB treatment	2.1	Psychosocial support is fundamental for people with TB to "feel secure in treatment"
		2.2	Nutritional supplementation is important for people with TB to be able to "fight against the disease"
3	Accessibility and acceptability are critical in designing social support interventions for people with TB	3.1	Support for people with TB can be prioritised by need – there is no one-size-fits-all solution
		3.2	Support should alleviate the burden of people with TB, not add to it
		3.3	Increasing access to TB diagnostic and preventive services is an important part of social protection

For peer review only

Theme 1: Existing financial safety nets are essential but could go further to support people with TB

Sub-theme 1.1: Enabling people with TB to meet medical costs is a priority for support

Participants were concerned about the financial burden of TB and viewed financial support as essential to reducing costs and ensuring good treatment outcomes. Most participants, particularly HCPs, thought that people with TB should not pay any medical costs during their treatment and noted this as their top priority for reducing costs and improving treatment outcomes. Participants valued provision of treatment by the NTP, but many people with TB reported paying different amounts depending on treatment location, treatment regimen or insurance status.

Participants described SHI as important, but difficult to access. Many people with TB reported that SHI registration was slow and expensive (particularly for households) and many were not registered at the right level. In fact, people with TB and HCPs alike reported that seeking care outside of the primary registered health facility was challenging. HCPs also noted difficulties with making claims, calculating payments and deductibles.

For peer review only - http://bmjopen.bmj.com/site/about/guidelines.xhtml

However, many participants prioritised SHI for reducing the costs of TB care. One male District TB Unit HCP summarised this view, saying *"We should focus on basic support. For example, health insurance is necessary, then testing"*. Overall, participants thought that uninsured people with TB should be provided with SHI, that SHI coverage or the NTP's provision should be expanded to cover more costs including diagnostic and confirmatory testing (including X-rays and GeneXpert), hospitalisation fees and supplements and that registration should be made simpler.

Sub-theme 1.2: Flexible financial support can give people with TB more agency

Many participants thought that cash transfers were important for mitigating out-of-pocket expenses and income loss and improving treatment outcomes. For people with TB in particular, cash transfers were a priority because they saw them as simple, quick and versatile, and could be used to solve upfront costs such as hospitalisation fees. One male participant who was successfully treated for DS-TB expressed that *"Cash, in general. Cash solves many things. It's simple"*. Further, cash transfers were seen by most as easier to implement than vouchers, easier to access than loans, and could give people with TB more independence to spend at their discretion. However, other people with TB spoke about a potential to misuse cash transfers (e.g. purchasing tobacco) and therefore thought that transport or nutrition vouchers were a better way to provide financial support.

People with TB also wanted support to find suitable employment to counter lost income. They indicated that career counselling, help with finding a new, less physically demanding job through connection with a job centre or job placement, or lending money to invest in their career would be beneficial. This was seen as more useful than vocational support, with one male participant who was successfully treated for DS-TB saying "*After training, there must be available jobs, or else, it's just a waste of time, it's very difficult to re-train*".

Theme 2: It is important to promote "physical and spiritual health" during TB treatment

Sub-theme 2.1: Psychosocial support is key for people with TB to "feel secure in treatment"

People with TB thought that financial support could also reduce worry and provide a sense of security during treatment, and thus improve mental health. This was summarised by one participant who was unsuccessfully treated for DS-TB who said that *"Sometimes, it is only a small part of a support package, but it provides them [the beneficiary] with something for their spirit- to help them make their best effort in their treatment. They feel better and cared for by society".*

All participants spoke about the negative impact that TB has on mental health. One female National/Provincial TB HCP described it as: *"There are patients who know they are depressed, bored, feel that they are not getting better, they are still tired from taking medication, the illness, they want to give up"*. More broadly, people with TB frequently referred to how pessimism can make it harder to recover and about the importance of feeling secure in their treatment.

As a result, many participants recognised a need for psychological, emotional and spiritual support to promote better treatment outcomes. People with TB described emotional support from friends and relatives as essential, but recognised a need for increased access to

For peer review only - http://bmjopen.bmj.com/site/about/guidelines.xhtml

a psychologist or psychiatrist, particularly for people with MDR-TB. Similarly, TB HCPs identified a need for psychological training given the scarcity of mental health specialists, as highlighted by this female National/Provincial TB HCP *"Psychiatrists are very, very rare. Even our [national-level] hospital does not have any. So sometimes, we see our patients and sincerely share from our hearts and our thoughts. But in terms of psychological-expertise, we have no specialization in psychology"*. However, some people with DS-TB reported no psychological problems and were reluctant to engage with proposed support.

Participants also thought that patient support groups could help people with TB to learn from one another and encourage optimism about treatment. This was summarised by one female participant with MDR-TB as *"When we undergo the treatment, we talk to people who don't have the disease, they won't understand us… But it's easier to talk to people who feel the same way as we do"*. Participants thought that these groups should be led by experts, whether that be HCPs, mental health specialists, or others with TB.

For many participants, particularly people with MDR-TB, comprehensive TB education and guidance from HCPs about TB treatment was an important part of feeling secure in their treatment, for example by helping them to manage side effects. People with TB thought that information should be provided by doctors at the start of treatment and available throughout. One participant with MDR-TB suggested that specialist pharmacists would be able to answer questions about side-effects of medicines. HCPs also suggested using leaflets, recorded messages and public media to share information about TB prevention and treatment.

Sub-theme 2.2: Nutritional supplementation is viewed as important for people with TB to be able to "fight against the disease"

Most participants thought that good nutrition and rest were essential to recovery and a priority for support, particularly for people with MDR-TB. One HCP at a District TB Unit summarised that *"tuberculosis is exhausting. If you are malnourished, you need energy, fibre, and vitamins".* Some participants thought that people with TB should be supported directly with food but many considered cash to be more helpful.

Participants also thought that supplements and tonics were essential for controlling side effects (e.g. through detoxifying the liver). All participants thought that they should be included in a support package rather than paid out-of-pocket. One National/Provincial TB HCP highlighted how costs can accumulate throughout treatment by explaining that *"Some patients don't have money to buy them, so they just receive the TB medications, no supplements. Then they have liver and kidney issues and get hospitalised. Then, the cost of liver and kidney detox would be many times higher than the cost of liver supplements they should have paid for in the beginning".*

Theme 3: Accessibility and acceptability are critical in designing social support interventions for people with TB

Sub-theme 3.1: Support for people with TB can be prioritised by need – there is no one-size-fits-all solution

For peer review only - http://bmjopen.bmj.com/site/about/guidelines.xhtml

Participants were asked who should be prioritised to receive each type of support and when it should be delivered. Some participants in each group thought that all people with TB should be supported equally, reasoning that prioritisation would cause jealousy, and that it should last throughout treatment. However, most participants thought that support for people with TB should be tailored to their individual needs.

Participants thought that people struggling financially or with more severe disease had greater needs for support and should therefore be prioritised. For example, one male participant successfully treated for DS-TB suggested that *"TB patients should be all supported, dividing by severity or circumstances, the poor or persons living in difficult situations, second treatment or drug resistance."*. Similarly, when prompted to prioritise when support was given, many people with TB suggested it be given during the intensive phase of treatment. On the other hand, many National/Provincial TB HCPs were explicit that support should be highly specific, with transport vouchers according to distance travelled or financial support according to days of hospitalisation.

Sub-theme 3.2: Support should alleviate the burden of people with TB, not add to it

Speaking from past experiences, TB HCPs raised concerns that defining eligibility criteria for support would create overly burdensome registration systems that impede access. One District TB HCP summarised that *"If you offer, but in the end you can't support due to the inflexible procedures... You support nobody."*

People with TB were also concerned with the practicality of proposed support options. Transport vouchers were viewed positively because they help people attend appointments and adhere to treatment. However, delivering food was viewed as a waste of time and resources. Similarly, people with TB viewed vocational training as impractical with one male participant who was successfully treated for DS-TB saying *"As old people have a certain job, it's hard to change to another. It's okay to just introduce an easier job for them, vocational training is not feasible."*

Additionally, participants wanted support to be convenient and accessible. They viewed current support as insufficient or inaccessible due to inflexible procedures or inadequate publicity. People with DS-TB viewed patient support groups positively but were reluctant to participate due to the time commitment and fears over re-infection. District TB HCPs had concerns about adding to their own overburdened workload by running these groups in person. Participants thought that online support groups would increase access to support in the context of limited staff resources and suggested using online forums on social media platforms to ask questions and share information. However, they had concerns about the accessibility of online support for older people.

Similarly, people with MDR-TB and National/Provincial TB HCPs viewed remote delivery of medication and online consultations as an additional way to access support when necessary such as during periods of intense COVID-19 restrictions but people with TB did not want to replace in-person appointments entirely.

For peer review only - http://bmjopen.bmj.com/site/about/guidelines.xhtml

Sub-theme 3.3: Increasing access to TB diagnostic and preventive services is an important part of social protection

Many participants thought that any support given should extend beyond the individual. Access to testing and preventive treatment for families affected by TB was prioritised by many people with TB and National/Provincial TB HCPs as an important way to reduce onwards transmission and reduce costs. People with TB suggested that their families be provided with home testing kits or transport vouchers to enable testing without incurring costs. One individual with MDR-TB highlighted the potential socio-protective effects of doing so, saying *"It's good for my family to go to the doctor to get screened… now there's a support system, it'll help with the expenses… If they go for screening, the disease is detected early, good for them. If you don't do it until it's severe, treatment will be more difficult."*.

Discussion

This study demonstrates that people with DS- and MDR-TB and TB HCPs perceive a wide range of social support and social protection measures to be important for reducing costs and improving TB treatment outcomes. People with TB face significant health, economic and psychosocial challenges during their treatment (2,5,6,18, 31) and their priorities for the design of supportive interventions reflect these. In keeping with a framework for transformative social protection (Figure 1) (30), our participants collectively identified a need for support that extends beyond financial risk protection to include a broader collection of promotive, protective and transformative interventions and psychosocial support.

For peer review only

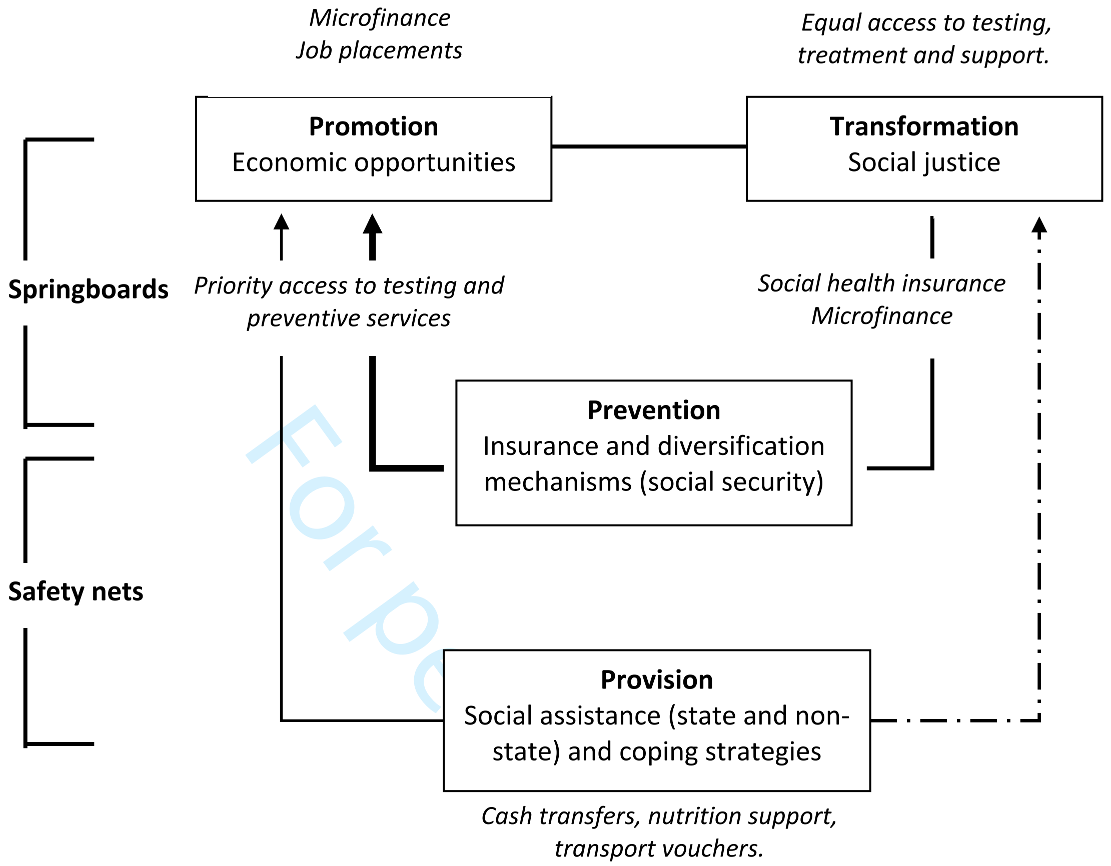

Figure 1: A conceptual framework for social protection, modified from Sabates-Wheeler and Devereux (30). Thicker lines indicate stronger relationships. The dotted line indicates where measures could have counter-productive effects on social transformation. The top half of the diagram represents policies that can facilitate movement out of poverty (springboards), while the bottom half represents those that alleviate and prevent deprivation (safety nets). Examples relevant to TB in italics.

Universal health coverage, including access to health insurance, is a key part of the WHO's End TB Strategy. In this study, many HCPs prioritised SHI to prevent people with TB from accruing catastrophic costs. However, they also recognised challenges with its implementation, reflecting recent findings in this setting (Forse, Yoshino, Nguyen et al. 2023). In Viet Nam, catastrophic costs remain high despite the provision from the NTP, high coverage of SHI and subsidies from the Global Fund for DR-TB (16, 23). SHI, or this subsidy, should therefore be extended to cover all treatment costs for all people with TB. In fact, the government of Viet Nam is aiming to achieve 100% coverage of SHI by 2025 (17) and has recently announced that TB care will soon be included under the SHI system (32). The implications of this change on people with TB are currently unclear. However, participants also reported high non-medical costs and lost income in line with previous studies (2,18) so, as recognised by the NTP (19), supportive interventions for people with TB in urban Viet Nam should extend beyond SHI.

Cash transfers are already a well-established part of the global response HIV and evidence is mounting for their positive impact on reducing costs and improving treatment outcomes for

For peer review only - http://bmjopen.bmj.com/site/about/guidelines.xhtml

people with TB (33). In fact, a recent scoping review found that conditional cash transfers were among the most common forms of social protection for TB-affected households (13). For example, cash transfers for people with TB in Peru led to improved treatment success and decreased likelihood of incurring catastrophic costs (34, 35). Therefore, it is perhaps unsurprising that all participant groups in this study identified cash transfers as a priority for both improving outcomes and reducing costs. Cash transfers are already provided for poor or uninsured people with TB by Viet Nam's government and for people with DS-TB in certain regions of Ha Noi and Ho Chi Minh City under donor projects (36), so expanding to more people with TB may bring wider benefits.

There is an ongoing debate regarding whether cash, vouchers or in-kind support are more effective (37). Our study highlighted that cash transfers may be preferable as they give people with TB more agency and are simple to manage, as well as being more cost-effective (37). However, direct non-medical costs make up a large share of costs during TB treatment (6), so targeted provision measures, such as transport or nutrition vouchers, can provide a 'safety net' to avoid the incurrence and exacerbation of catastrophic costs. Our study demonstrates that alleviating transport costs is important for people with TB both for mitigating costs and improving treatment outcomes, complementing previous findings that transport vouchers can improve treatment adherence and success rates (38, 39). Participants also viewed nutritional and supplement support as key to managing side effects and associated costs. Nutrition or food support programs for people with TB are common (13). However, while there is some evidence that food assistance can improve treatment adherence (40), the evidence for the clinical benefit of nutritional supplementation (41) or of traditional supplements (42) is limited. Consequently, cash transfers and transport vouchers may be a better way to address non-medical costs, with further research required around the benefits of nutritional and traditional supplements.

The indirect cost of lost productivity during treatment is a significant burden for people with TB in Viet Nam (18) and other high TB burden settings (6), particularly considering the chronic impact of income loss (43). Therefore, promotive interventions that support people with TB to establish a sustainable livelihood can provide a 'springboard' out of poverty (3,30) as well as contributing to improved TB treatment outcomes (44). In this study we found that job placements or career counselling were preferred over vocational training and could be an effective way to support people with TB, particularly breadwinners and younger people, to re-enter the workforce. However, there must be opportunities for employment available after such interventions. Given the adverse impact of COVID-19 on employment in urban Viet Nam (45), enabling employment for people with TB may be more important than ever.

Social protection is one part of a broader set of efforts needed to contain TB (8). Participants in our study also emphasised the importance of social support, particularly psychological support. People with TB, particularly MDR-TB, often experience psychological distress during treatment (14,31,46) and previous studies show that psychological support can lead to improved treatment outcomes (14,34). Integrating psychological support into existing care by training TB HCPs, as suggested by our participants, has been shown to be effective in another context (47). Informal online patient support groups could also promote wider

For peer review only - http://bmjopen.bmj.com/site/about/guidelines.xhtml

access to psychosocial support and TB education by allowing people with TB to learn from one another, noting potential difficulties for older people, and may become more acceptable following the COVID-19 pandemic. In line with the framework for transformative social protection, which emphasises both financial and social aspects of vulnerability (30), there is evidence that combined psychological and financial interventions are more effective at improving treatment outcomes than financial support alone (14,48). This could therefore improve supportive interventions for people with TB in urban Viet Nam.

Our participants highlighted the importance of accessibility of TB services and support in line with a recent scoping review (13), both for people with TB and their families.
One way in which this could be achieved is through active case finding (ACF)to identify unidentified persons with TB. This is especially important in Viet Nam given that just 46% of estimated new TB cases in 2021 were treated (1). Viet Nam's NTP has recognised the importance of ACF as it can reduce transmission and improve health outcomes over time. Furthermore, there is recent evidence from Ho Chi Minh City that it can reduce the incurrence of catastrophic costs and can reach more marginalised individuals, increasing equity in TB care (49). ACF could therefore be considered a key part of a wider package of social protection measures in this context and contribute to achieving the End TB goals (7).

Finally, our study suggests that supportive interventions for people with TB could be prioritised to make effective use of resources by allocating based on individual need and targeting the intensive phase of treatment when costs are highest. Differentiated service delivery, in which services are tailored to individual needs, is already a key aspect of person-centred HIV care that has been shown to improve access to and uptake of testing and treatment (50). A similar approach could be taken to allocating packages of support for people with TB, learning from experiences with HIV to ensure that any eligibility criteria did not create barriers to accessing support. However, modes of differentiating support are highly context dependent (33, 50) and further research is required to identify optimal approaches for people with TB in Viet Nam.

Strengths and limitations
A key strength of this study is the inclusion of a diverse group of participants, enhancing trustworthiness and validity and ensuring that the resulting recommendations take their views into account. There was broad consensus between participants, suggesting robustness of the findings. Interviews were conducted in the NGO offices rather than in the health facility to mitigate social desirability bias.

A limitation is that this study was conducted solely in urban settings in Viet Nam, therefore the findings may not be generalisable to rural settings within Viet Nam or to other high burden countries. The needs of people with TB may have shifted since data collection given the widespread impact of COVID-19 on health service utilisation and economic wellbeing (52, 53) as well as changes to financing for TB (32).

Further, the analysis was conducted on translated transcripts which may have influenced their interpretation (51). However, the study team took a rigorous approach to transcription and translation and there was close collaboration between Vietnamese- and non-Vietnamese-speaking team members to limit the effect of ethnocentrism on the analysis.

For peer review only - http://bmjopen.bmj.com/site/about/guidelines.xhtml

Implications and conclusions

Our study highlights that a broad range of social protection and social support measures are required to meet the needs of people with TB in urban Viet Nam. While Viet Nam has begun to implement social support for people with TB, we found that interventions that extend beyond the health system are needed to better protect people with TB against costs and to improve treatment outcomes.

In conjunction with prior research, this study could play an important role in the design of supportive interventions for people with DS- and MDR-TB. Within urban Viet Nam, it could guide how government funds such as PASTB support people with TB with recommendations for actions outlined in Table 4. To expand on this, future research could explore the optimal operational design of interventions, for example by evaluating whether online support groups or psychological training for HCPs are the best way to support people with TB with their mental health.

More broadly, this study suggests that coordinated action from biomedical, social and economic sectors is needed to support people with TB and other diseases of poverty in Viet Nam and other high burden countries. This can also have a positive impact on wider development; TB affects many people in their most productive years, so supporting them to break the cycle of poverty and disease could enable economic growth and sustainable development. Overall, this study demonstrates that further action on the social determinants of TB is needed in the global effort towards containing TB and eliminating catastrophic costs.

Table 4: Recommendations for action from this study based on participant responses.

**To increase access to psychological support:**
Establish online peer support forums using platforms such as messenger applications (such as WhatsApp or Zalo) involving people with Tuberculosis (TB) and TB Healthcare Practitioners (HCPs) to act as an information-sharing platform and enable patients to report and receive assistance for side-effects
Increase availability of trained psychologists and psychiatrists where possible, or provide psychological training for TB HCPs, particularly for people with multi-drug resistant TB (MDR-TB)
Ensure comprehensive TB education to help people with TB manage their treatment and reduce worry, including guided counselling sessions between people with TB and TB doctors at the beginning of treatment as standard of care
**To prevent the accumulation of catastrophic costs:**
Establish, or link people with TB to existing, career counselling services or job placements
Provide cash transfers to people with TB to be spent at their discretion
Ensure all people with TB are covered by Viet Nam's national Social Health Insurance scheme, and plug gaps in coverage for those who are
**To increase access to TB care and support:**
Increase access to testing and treatment for TB infection and ensure all contacts of people with TB are tested for TB
Continue and extend existing transport and nutritional support, particularly for people with MDR-TB
Differentiate all support according to multidimensional need, considering economic status and severity of disease as appropriate

For peer review only

Contributorship statement

IS co-developed the research questions, led data analysis (developing the coding framework and identifying themes), drafted and revised the manuscript. RF conceptualised the study, designed topic guides, oversaw data collection, supported and verified analysis, and reviewed the manuscript. She is the guarantor for the paper. KSA conceptualised the study, designed topic guides, co-developed the research questions. TTN conceptualised the study, oversaw data collection, advised on and reviewed the manuscript. LN, THYP, HN, AJC, LNQV, NN, AK, JC, MP, LB, KL, HBN, NVN edited and reviewed the manuscript. SA co-developed the research questions and the coding framework, supported and verified analysis and reviewed the manuscript.

Acknowledgements

Our special thanks go to the participants in this study for their invaluable contributions to this study. We are particularly grateful to the people with TB for sharing their personal experiences of illness and hope that this study will have a positive impact on people with TB in the future.

We would like to thank the individuals who conducted interviews including: Nham Thi Yen Ngoc, Tran Thi Ngan, and Bui Thi Huyen. Special thanks to the individuals who completed transcriptions, translations and the checking of these processes, including Nguyen Thi Cam Van, Truong Thi Thuy Dung, Nguyen Thi Xuan, Phan Khanh Hang and Huynh Thi Kim Ngan.

We appreciate the staff of the National Lung Hospital, Ha Noi Lung Hospital, and Pham Ngoc Thach Hospital who facilitated interviews for our research team. TB staff based in the following districts of Ha Noi assisted with participant recruitment: Ba Dinh, Dong Da, Hai Ba Trung, Hoan Kiem, and Hoang Mai. In Ho Chi Minh City, we would like to thank staff from five districts who assisted with recruitment including: Binh Chanh, Go Vap, Thu Duc and Districts 6 & 8.

This study was made possible by the generous support of the American people through USAID. The contents are the responsibility of the listed authors, and do not necessarily reflect the views of USAID or the United States Government.

Competing interests: None declared.

Data Availability Statement: Data are available upon reasonable request.

Funding sources: This study was supported by two funders. Interviews, transcriptions and translations among people with DS-TB were funded by the United States Agency for International Development (USAID) Fixed Award No. 72044020FA00001, through the Erase TB Activity. DR-TB interviews were conducted with support from the Stop TB Partnership's

For peer review only - http://bmjopen.bmj.com/site/about/guidelines.xhtml

TB REACH initiative by Global Affairs Canada grant number CA-3-D000920001 https://w05.international.gc.ca/projectbrowserbanqueprojets/projectprojet/details/d000920001. The funders had no role in study design, data collection and analysis, decision to publish, or preparation of the manuscript.

Ethical considerations: This study was conducted in strict adherence with the Declaration of Helsinki. Ethical approvals were obtained from Ha Noi University School of Public Health (issued on July 17, 2020; IRB #300/2020/YTCC-HD3) and from Pham Ngoc Thach Hospital (issued on September 16, 2020; IRB # 1225/PNT-HĐĐĐ).

Informed consent was obtained prior to study initiation. At the start of each FGD or KII, a patient information sheet (PIS) and consent form were provided and explained by the moderators. All participants signed the informed consent forms before data collection began and were free to withdraw consent and leave the study at any time.

In terms of infection prevention and control measures, most participants were cured or had completed treatment and participants in the unsuccessful treatment group had been on anti-TB medications for more than one month. Many participants wore masks and hand sanitiser was provided to all participants on entry.

A data-sharing agreement was reached between Friends for International TB Relief (one of the local NGO partners) and the Karolinska Institutet. FIT collected data directly or obtained it through collaboration agreements with local collaborating NGO partners. All data were pseudonymised prior to sharing and stored on a secure, password-protected online platform at Karolinska Institutet.

References

1. World Health Organisation. Global Tuberculosis Report 2022 [Internet]. Geneva; 2022 [cited 2022 Oct 30]. Available from: https://www.who.int/teams/global-tuberculosis-programme/tb-reports/global-tuberculosis-report-2022
2. Lönnroth K, Glaziou P, Weil D, Floyd K, Uplekar M, Raviglione M. Beyond UHC: Monitoring Health and Social Protection Coverage in the Context of Tuberculosis Care and Prevention. PLoS Med [Internet]. 2014 Sep 1 [cited 2022 Jan 30];11(9):e1001693. Available from: https://journals.plos.org/plosmedicine/article?id=10.1371/journal.pmed.1001693
3. Hargreaves JR, Boccia D, Evans CA, Adato M, Petticrew M, Porter JDH. The Social Determinants of Tuberculosis: From Evidence to Action. Am J Public Health [Internet]. 2011 Apr [cited 2022 Apr 30];101(4):654. Available from: /pmc/articles/PMC3052350/
4. Waitt CJ, Squire SB. A systematic review of risk factors for death in adults during and after tuberculosis treatment. International Journal of Tuberculosis and Lung Disease. 2011 Jul;15(7):871–85.
5. Mauch V, Bonsu F, Gyapong M, Awini E, Suarez P, Marcelino B, et al. Free tuberculosis diagnosis and treatment are not enough: patient cost evidence from three continents. Int J Tuberc Lung Dis [Internet]. 2013 Mar 1 [cited 2022 May 2];17(3):381–7. Available from: https://pubmed.ncbi.nlm.nih.gov/23407227/

For peer review only

6. Tanimura T, Jaramillo E, Weil D, Raviglione M, Lönnroth K. Financial burden for tuberculosis patients in low- and middle-income countries: a systematic review. Eur Respir J [Internet]. 2014 [cited 2022 Apr 28];43(6):1763. Available from: /pmc/articles/PMC4040181/

7. Tran PB, Hensing G, Wingfield T, Income security during public health emergencies: the COVID-19 poverty trap in Vietnam, *BMJ Global Health* 2020;5:e002504

8. World Health Organisation. The end TB strategy [Internet]. Geneva; 2015 [cited 2022 Apr 28]. Available from: https://www.who.int/publications/i/item/WHO-HTM-TB-2015.19

9. Siroka A, Ponce NA, Lönnroth K. The impact of social protection on tuberculosis rates: a global analysis. Lancet Infect Dis [Internet]. 2016 Apr 1 [cited 2022 Apr 28];16(4):473. Available from: /pmc/articles/PMC4834192/

10. Carter DJ, Glaziou P, Lönnroth K, Siroka A, Floyd K, Weil D, et al. The impact of social protection and poverty elimination on global tuberculosis incidence: a statistical modelling analysis of Sustainable Development Goal 1. Lancet Glob Health [Internet]. 2018 May 1 [cited 2022 Jan 30];6(5):e514–22. Available from: https://pubmed.ncbi.nlm.nih.gov/29580761/

11. Boccia D, Hargreaves J, Lönnroth K, Jaramillo E, Weiss J, Uplekar M, et al. Cash transfer and microfinance interventions for tuberculosis control: review of the impact evidence and policy implications. Int J Tuberc Lung Dis [Internet]. 2011 Jun [cited 2022 Apr 28];15 Suppl 2(Suppl 2). Available from: https://pubmed.ncbi.nlm.nih.gov/21740658/

12. Richterman A, Steer-Massaro J, Jarolimova J, Nguyen LBL, Werdenberg J, Ivers LC. Cash interventions to improve clinical outcomes for pulmonary tuberculosis: systematic review and meta-analysis. Bull World Health Organ [Internet]. 2018 Jul 1 [cited 2022 May 4];96(7):471. Available from: /pmc/articles/PMC6022611/

13. Todd H, Hudson M, Grolmusova N, Kazibwe J, Pearman J, Skender K, Tran PB, Boccia D, Shete PB, Wingfield T. Social Protection Interventions for TB-Affected Households: A Scoping Review. American Journal of Tropical Medicine and Hygiene [Internet]. 2023. [cited 2023 Apr 4]. Available from: https://doi.org/10.4269/ajtmh.22-0470

14. van Hoorn R, Jaramillo E, Collins D, Gebhard A, van den Hof S. The Effects of Psycho-Emotional and Socio-Economic Support for Tuberculosis Patients on Treatment Adherence and Treatment Outcomes – A Systematic Review and Meta-Analysis. PLoS One [Internet]. 2016 Apr 1 [cited 2022 Apr 28];11(4). Available from: /pmc/articles/PMC4849661/

15. World Health Organization. WHO global lists of high burden countries for TB, multidrug/rifampicin-resistant TB (MDR/RR-TB) and TB/HIV, 2021-20205. Geneva;2021. Available from: https://www.who.int/news/item/17-06-2021-who-releases-new-global-lists-of-high-burden-countries-for-tb-hiv-associated-tb-and-drug-resistant-tb

16. Nguyen HV, Tiemersma EW, Nguyen HB, Cobelens FGJ, Finlay A, Glaziou P, et al. The second national tuberculosis prevalence survey in Vietnam. PLoS One [Internet]. 2020 Apr 1 [cited 2022 Mar 20];15(4):e0232142. Available from: https://journals.plos.org/plosone/article?id=10.1371/journal.pone.0232142

17. Thi Mai Oanh T, Khanh Phuong N, Anh Tuan K. Sustainability and Resilience in the Vietnamese Health System Sustainability and Resilience in the Vietnamese Health System Sustainability and Resilience in the Vietnamese Health System [Internet]. 2021 [cited 2022 Mar 8]. Available from: https://weforum.org/phssr

18. Nhung N v., Hoa NB, Anh NT, Anh LTN, Siroka A, Lönnroth K, et al. Measuring catastrophic costs due to tuberculosis in Viet Nam. International Journal of Tuberculosis and Lung Disease. 2018 Sep 1;22(9):983–90.

For peer review only - http://bmjopen.bmj.com/site/about/guidelines.xhtml

19. Hoa NB, Nhung N v. National tuberculosis patients cost survey: research findings lead to change in policy and practice, Viet Nam. Public Health Action [Internet]. 2019 Jul 23 [cited 2022 May 2];9(2):50. Available from: /pmc/articles/PMC6645445/
20. Tong A, Sainsbury P, Craig J. Consolidated criteria for reporting qualitative research (COREQ): A 32-item checklist for interviews and focus groups. International Journal for Quality in Health Care. 2007 Dec;19(6):349–57.
21. United States Agency for International Development. Fact Sheet: Erase TB Project | Vietnam | U.S. Agency for International Development [Internet]. 2020 [cited 2022 May 11]. Available from: https://www.usaid.gov/vietnam/documents/fact-sheet-erase-tb-project
22. Friends for International TB Relief. Friends for International TB Relief (FIT) [Internet]. [cited 2022 May 11]. Available from: https://tbhilfe.org/
23. Phát triển bền vững BHYT toàn dân [Sustainable development of universal health insurance] [Internet]. Bao Hiem Xa Hoi [Vietnam Social Security]. 2019 [cited 2022 Aug 26]. Available from: https://baohiemxahoi.gov.vn/congkhai/Pages/so-lieu-thong-ke-nganh.aspx?CateID=130&ItemID=12916
24. Thủ tục hành chính: Đăng ký đóng, cấp thẻ BHYT đối với người chỉ tham gia BHYT [Details of Small Administrative Procedures: Register to pay and issue health insurance cards for people only participating in health insurance] [Internet]. Bao Hiem Xa Hoi [Vietnam Social Security]. [cited 2023 Mar 17]. Available from: https://baohiemxahoi.gov.vn:443/thu-tuc-hanh-chinh/Pages/default.aspx?ItemID=73
25. Vuong Q-H, La V-P, Nguyen M-H, Nguyen T-HT, Ho M-T. Good budget or good care: The dilemma of social health insurance in Vietnam. *SAGE Open Medicine*. 2021;9. doi:10.1177/20503121211042512
26. Malterud K, Siersma VD, Guassora AD. Sample Size in Qualitative Interview Studies: Guided by Information Power. Qual Health Res [Internet]. 2016 Nov 1 [cited 2022 Mar 8];26(13):1753–60. Available from: https://journals-sagepub-com.proxy.kib.ki.se/doi/full/10.1177/1049732315617444
27. Braun V, Clarke V. Using thematic analysis in psychology. Qual Res Psychol [Internet]. 2006 [cited 2022 Jan 30];3(2):77–101. Available from: https://www.tandfonline.com/action/journalInformation?journalCode=uqrp20
28. Braun V, Clarke V. Reflecting on reflexive thematic analysis. Qual Res Sport Exerc Health [Internet]. 2019;11(4):589–97. Available from: https://www.tandfonline.com/action/journalInformation?journalCode=rqrs21
29. Dedoose version 9.0.17, web application for managing, analysing and presenting qualitative and mixed methods research data. Los Angeles, CA: SocioCultural Research Consultants, LLC; 2021.
30. Sabates-Wheeler R, Devereux S. Social protection for transformation. IDS Bull. 2007 May;38(3):23–8.
31. Thomas BE, Shanmugam P, Malaisamy M, Ovung S, Suresh C, Subbaraman R, et al. Psycho-Socio-Economic Issues Challenging Multidrug Resistant Tuberculosis Patients: A Systematic Review. PLoS One [Internet]. 2016 Jan 1 [cited 2022 May 3];11(1):e0147397. Available from: https://journals.plos.org/plosone/article?id=10.1371/journal.pone.0147397
32. Tran VT. Circular No. 36/2021/TT-BYT dated December 31, 2021 on Medical examination and treatment, and payment for medical examination and treatment costs covered by health insurance relating to tuberculosis examination and treatment [Internet]. 36/2021/TT-BYT Dec 31, 2021. Available from: https://hethongphapluat.com/circular-no-36-2021-tt-byt-dated-december-31-2021-on-medical-examination-and-treatment-and-payment-for-

For peer review only

For peer review only - http://bmjopen.bmj.com/site/about/guidelines.xhtml

medical-examination-and-treatment-costs-covered-by-health-insurance-relating-to-tuberculosis-examination-and-treatment.html
33. Boccia D, Pedrazzoli D, Wingfield T, Jaramillo E, Lönnroth K, Lewis J, et al. Towards cash transfer interventions for tuberculosis prevention, care and control: key operational challenges and research priorities. BMC Infect Dis [Internet]. 2016 Jun 21 [cited 2022 Apr 28];16(1). Available from: /pmc/articles/PMC4915091/

34. Wingfield T, Tovar MA, Huff D, Boccia D, Montoya R, Ramos E, et al. A randomized controlled study of socioeconomic support to enhance tuberculosis prevention and treatment, Peru. Bull World Health Organ [Internet]. 2017 Apr 1 [cited 2022 May 4];95(4):270. Available from: /pmc/articles/PMC5407248/ (previously 43)
35. Wingfield T, Tovar MA, Huff D, Boccia D, Montoya R, Ramos E, Lewis JJ, Gilman RH, Evans CA. The economic effects of supporting tuberculosis-affected households in Peru. Eur Respir J. 2016 Nov;48(5):1396-1410. doi: 10.1183/13993003.00066-2016. Epub 2016 Sep 22. PMID: 27660507; PMCID: PMC5091496.
36. United States Agency for International Development. Fact Sheet: Erase TB Project | Vietnam | U.S. Agency for International Development [Internet]. 2020 [cited 2022 May 11]. Available from: https://www.usaid.gov/vietnam/documents/fact-sheet-erase-tb-project
37. Gentilini U. The Revival of the 'Cash versus Food' Debate New Evidence for an Old Quandary? [Internet]. 2016 [cited 2022 May 4]. Available from: http://econ.worldbank.org.
38. Ciobanu A, Domente L, Soltan V, Bivol S, Severin L, Plesca V, et al. Do incentives improve tuberculosis treatment outcomes in the Republic of Moldova? Public Health Action [Internet]. 2014 Oct 21 [cited 2022 Sep 19];4(Suppl 2):S59–63. Available from: https://pubmed.ncbi.nlm.nih.gov/26393100/
39. Lu H, Yan F, Wang W, Wu L, Ma W, Chen J, et al. Do transportation subsidies and living allowances improve tuberculosis control outcomes among internal migrants in urban Shanghai, China? Western Pac Surveill Response J [Internet]. 2013 Jan 1 [cited 2022 May 10];4(1):19–24. Available from: https://pubmed.ncbi.nlm.nih.gov/23908951/
40. de Pee S, Grede N, Mehra D, Bloem MW. The enabling effect of food assistance in improving adherence and/or treatment completion for antiretroviral therapy and tuberculosis treatment: a literature review. AIDS Behav [Internet]. 2014 Oct 1 [cited 2022 May 4];18 Suppl 5:531–41. Available from: https://pubmed.ncbi.nlm.nih.gov/24619602/
41. Grobler L, Nagpal S, Sudarsanam TD, Sinclair D. Nutritional supplements for people being treated for active tuberculosis. Cochrane Database Syst Rev. 2016 Jun 29;2016(6):CD006086. doi: 10.1002/14651858.CD006086.pub4. PMID: 27355911; PMCID: PMC4981643.
42. Liu Q, Garner P, Wang Y, Huang B, Smith H. Drugs and herbs given to prevent hepatotoxicity of tuberculosis therapy: systematic review of ingredients and evaluation studies. BMC Public Health. 2008 Oct 21;8:365. doi: 10.1186/1471-2458-8-365. PMID: 18939987; PMCID: PMC2576232.
43. Meghji J, Gregorius S, Madan J, Chitimbe F, Thomson R, Rylance J, et al. The long term effect of pulmonary tuberculosis on income and employment in a low income, urban setting. Thorax [Internet]. 2021 Apr 1 [cited 2022 May 4];76(4):387–95. Available from: https://pubmed.ncbi.nlm.nih.gov/33443228/
44. Vanleeuw L, Zembe-Mkabile W, Atkins S. "I'm suffering for food": Food insecurity and access to social protection for TB patients and their households in Cape Town, South Africa. Vousden G, editor. PLoS One [Internet]. 2022 Apr 26 [cited 2022 May 2];17(4):e0266356. Available from: /pmc/articles/PMC9041827/

For peer review only - http://bmjopen.bmj.com/site/about/guidelines.xhtml

45. General Statistics Office of Viet Nam. Report on the Covid-19 impacts on labour and employment situation in the third quarter of 2021 [Internet]. Ha Noi; 2021 Oct [cited 2022 Sep 13]. Available from: https://www.gso.gov.vn/en/data-and-statistics/2021/10/report-on-the-covid-19-impacts-on-labour-and-employment-situation-in-the-third-quarter-of-2021/
46. Long NH, Johansson E, Diwan VK, Winkvist A. Fear and social isolation as consequences of tuberculosis in VietNam: a gender analysis. Health Policy (New York). 2001 Oct 1;58(1):69–81.
47. Chalco K, Wu DY, Mestanza L, Muñoz M, Llaro K, Guerra D, et al. Nurses as providers of emotional support to patients with MDR-TB. Int Nurs Rev [Internet]. 2006 Dec [cited 2022 May 5];53(4):253–60. Available from: https://pubmed.ncbi.nlm.nih.gov/17083413/
48. Baral SC, Aryal Y, Bhattrai R, King R, Newell JN. The importance of providing counselling and financial support to patients receiving treatment for multi-drug resistant TB: mixed method qualitative and pilot intervention studies. BMC Public Health [Internet]. 2014 Jan 17 [cited 2022 May 5];14(1):46. Available from: /pmc/articles/PMC3898066/
49. Vo LNQ, Forse RJ, Codlin A, Dang HA, Truong V v., Nguyen LJ, et al. Socio-protective effects of active case finding on catastrophic costs from tuberculosis in Ho Chi Minh City, Viet Nam: a longitudinal patient cost survey. BMC Health Serv Res [Internet]. 2021 [cited 2022 Oct 24];21(1051). Available from: https://doi.org/10.1186/s12913-021-06984-2
50. Roy M, Bolton Moore C, Sikazwe I, Holmes CB. A Review of Differentiated Service Delivery for HIV Treatment: Effectiveness, Mechanisms, Targeting, and Scale. Vol. 16, Current HIV/AIDS Reports. 2019.
51. Smith HJ, Chen J, Liu X. Language and rigour in qualitative research: Problems and principles in analyzing data collected in Mandarin. BMC Medical Research Methodology [Internet]. 2008 Jul 10 [cited 2022 May 4];8(1):1–8. Available from: https://bmcmedresmethodol.biomedcentral.com/articles/10.1186/1471-2288-8-44
52. Moynihan R, Sanders S, Michaleff ZA, Scott AM, Clark J, To EJ, et al. Impact of COVID-19 pandemic on utilisation of healthcare services: a systematic review. BMJ Open [Internet]. 2021 Mar 1 [cited 2022 May 5];11(3):e045343. Available from: https://bmjopen.bmj.com/content/11/3/e045343
53. Tran BX, Nguyen HT, Le HT, Latkin CA, Pham HQ, Vu LG, et al. Impact of COVID-19 on Economic Well-Being and Quality of Life of the Vietnamese During the National Social Distancing. Frontiers in Psychology. 2020 Sep 11;11:2289.

For peer review only

Appendix A: Details of data collection

Participant group	Number of participants	Type	Location	Duration (minutes)
People with successfully treated DS-TB	5¶	FGD	Ha Noi (NGO office)	146
People with unsuccessfully treated DS-TB	4	FGD	Ha Noi (NGO office)	103
District TB Unit HCP	5	FGD	Ha Noi (NGO office)	91
People with successfully treated DS-TB	3	FGD	Ho Chi Minh City (NGO office)	138
People with unsuccessfully treated DS-TB	2	FGD	Ho Chi Minh City (NGO office)	113
People with MDR-TB	4	FGD	Ha Noi (Ha Noi Lung Hospital)	109
People with MDR-TB	6	FGD	Ho Chi Minh City (NGO office)	188
National/Provincial TB HCP	4*	KII	Ha Noi (Zoom)	46-90

¶ Indicates that at least one participant was accompanied by their caregiver
**Four separate KII were conducted.*

For peer review only

Appendix B: Topic guides for data collection

Focus Group Discussions (DTU and Provincial TB hospital staff)

Prior experiences of providing TB treatment support

- What existing social support resources are available for the TB patients that you treat?
  - How do you link your patients to existing resources?

Barriers to TB treatment

- From your experience, what is the biggest obstacle that prevents patients from completing treatment?
- What are the main factors that cause catastrophic costs or poverty during TB treatment?

Types of social support

- If you could design any intervention to support the TB patients you treat, what would be provided?

Social health insurance

- Which types of TB patients should receive health insurance? Why?
- Who should provide SHI to TB patients?
- How effective is SHI at alleviating the costs of TB care?

TB Education

- On what types of information should TB patients receive counselling and education?
- When should this information be given during the duration of treatment?
- If you were given any teaching aids to improve the patient counselling, what would those teaching aids look like?

Nutritional support

- What types of food are needed by TB patients?
- What types of supplements are needed during TB treatment?
- Which types of patients should be offered nutritional support, if it is offered?
- For how long do TB patients need nutritional support?
  - Prompt: during the intensive phase or throughout all of treatment?
- What are the benefits of providing food and/or supplements?
- Do you see any drawbacks to the provision of food and/or supplements?

For peer review only

Cash transfers

- Which types of TB patients should receive the cash transfer? Why?
- For how long should cash be given to TB patients?
- What are the benefits and drawbacks of cash transfers compared to other types of social support?
- How should cash be spent?
- How should cash not be spent?

Loans

- What was your need for credit during TB care?
- How do you feel about providing loans to TB patients? Whose role should it be to provide loans?
- How much credit should be given?
- How should TB patients spend the loan money?
- How would loans address the needs of TB patients?
- What should the payment plan consist of?
- How should we collect on the loan?

Transport vouchers

- How do you feel about transportation vouchers being given to TB patients?
- How do transport vouchers address the needs of TB patients?
- Which types of patients should be offered transport vouchers, if they are offered?
- How willing do you feel TB patients would be to participate in a project that provided transport vouchers?
- What challenges do you foresee in providing transport vouchers to TB patients?

Patient support groups

- What is your opinion of TB patient support groups?
- Which types of TB patients would benefit the most from patient support groups?
- What types of benefits would TB patients gain from a TB support group?
- How willing do you feel TB patients would be to participate patient support groups?
- During which stage of TB treatment would a patient support group be most effective to support the patient?
- Who is the most effective type of person to lead the patient support group?

For peer review only

- Prompt: Peer-led, DTU-led or psychologist-led
- How would you feel about hosting a patient support group?

Access to a psychologist/ psychiatrist for DS-TB patients

- What types of psychological or psychiatric issues do your DS-TB patients face?
- What mental health support services do DS-TB patients require?
- What resources are currently available to them for psychological or psychiatric problems?
- How willing do you feel DS-TB patients would be to meet with a mental health professional during their treatment?
- How would better access to a mental health professional improve DS-TB treatment outcomes?

Priority access to testing and preventative therapy

- How can patient's families best be supported to go for TB testing?
- What barriers do families of people with TB face in accessing TB testing?
- What is the best way to improve LTBI treatment initiation rates among TB contacts?
- What barriers do families of people with TB face in accessing LTBI testing and treatment?

Prioritization to improve treatment outcomes

- Following this discussion, what do you feel is the most important type of support for TB patients to improve their treatment outcomes? Why?
- What are the second and third most important types of support for TB treatment?

Prioritization to reduce catastrophic costs

- What do you feel is the most important type of support for TB patients to reduce catastrophic costs? Why?
- What are the second and third most important types of support to reduce patient costs?

Is there anything else you would like to tell us?

Focus Group Discussions (Current/Former TB patients)

Experiences on TB treatment

- When you were on TB treatment, what types of challenges did you face?
- What types of support did you need? Why?

For peer review only

Barriers to TB treatment

- From your experience, what is the biggest obstacle that prevents patients from completing treatment?
- What are the main factors that cause catastrophic costs or poverty during TB treatment?

Types of support

- If you could design any type of program to help TB patients, not thinking about the cost, what would that program include?

<u>Social health insurance</u>

- Which types of TB patients should receive health insurance? Why?
- Who should provide SHI to TB patients?
- How effective is SHI at alleviating the costs of TB care?

<u>Education</u>

- On what types of information should TB patients receive counselling?
- When should this information be given during the duration of treatment?
- Thinking back the to the counselling you received, if you could change anything about this counselling, what would you change?

<u>Nutritional support</u>

- What types of food are needed by TB patients?
- What types of supplements are needed during TB treatment?
- Which types of patients should be offered nutritional support, if it is offered?
- For how long do TB patients need nutritional support?
  - Prompt: during the intensive phase or throughout all of treatment?
- What are the benefits of providing food and/or supplements?
- Do you see any drawbacks to the provision of food and/or supplements?

<u>Cash transfers</u>

- Which types of TB patients should receive the cash transfer? Why?
- For how long should cash be given to TB patients?
- What are the benefits and drawbacks of cash transfers compared to other types of social support?

For peer review only

- How should cash be spent?
- How should cash not be spent?

Vocational training

- How helpful do you feel vocational training would be to TB patients?
    - Prompt: During treatment vs. after treatment
    - Prompt: short-term vs. long-term benefits
- Which types of patients should be offered vocational training, if it is offered?
- How willing do you feel TB patients would be to participate?
- What would be the benefits of vocational training to TB patients?
- Which barriers would TB patients face completing the vocational training?

Transport vouchers

- How do you feel about transportation vouchers being given to TB patients?
- How do transport vouchers address the needs of TB patients?
- Which types of patients should be offered transport vouchers, if they are offered?
- How willing do you feel TB patients would be to participate in a project that provided transport vouchers?
- What challenges do you foresee in providing transport vouchers to TB patients?
- What are the benefits of transport vouchers compared to cash payments?

Patient support groups

- What is your opinion of TB patient support groups?
- What types of challenges do patient support groups best address?
- What issues should patient support groups discuss?
- Which types of TB patients would benefit the most from patient support groups?
- What types of benefits would TB patients gain from a TB support group?
- How willing do you feel TB patients would be to participate patient support groups?
- During which stage of TB treatment would a patient support group be most effective to support the patient?
- Who is the most effective type of person to lead the patient support group?
    - Prompt: Peer-led, DTU-led or psychologist-led
- How would you feel about hosting a patient support group?

Access to a psychologist/ psychiatrist for DS-TB patients

For peer review only

- What mental health support services did you require during your treatment?
- What resources were available to you for psychological or psychiatric problems, if any?
- How willing do you feel DS-TB patients would be to meet with a mental health professional during their treatment?
- How would access to a mental health professional improve DS-TB treatment outcomes?

Access to testing and preventative therapy

- How can patient's families best be supported to go for TB testing?
- What barriers do families of people with TB face in accessing TB testing?
- What is the best way to improve LTBI treatment initiation rates among TB contacts?
- What barriers do families of people with TB face in accessing LTBI testing and treatment?

Prioritization to improve treatment outcomes

- Following this discussion, what do you feel is the most important type of support for TB patients to improve their treatment outcomes? Why?
- What are the second and third most important types of support for TB treatment?

Prioritization to reduce catastrophic costs

- What do you feel is the most important type of support for TB patients to reduce catastrophic costs? Why?
- What are the second and third most important types of support to reduce patient costs?

Is there anything else you would like to tell us?

For peer review only

KIIs (National and Provincial TB staff)

Barriers

- From your experience, what is the biggest obstacle that prevents patients from completing treatment?
- What are the main factors that cause catastrophic costs or poverty during DR-TB treatment?

Prior experiences of providing treatment support to people with DR-TB

- What existing support resources are available for the DR-TB patients that you treat?
    - How do you link your patients to existing resources?
    - How effective do you find these sources to be?
        - Describe the gaps in this support
        - How adequate do you find this support to be?
        - How easy or difficult is it for people with DR-TB to obtain economic or psychological support?
- Which groups of people with DR-TB require economic and psychological support?

Intervention design

- If you could design any intervention to support DR-TB patients, what would be provided?
    - Why would this be provided?
    - Who should receive it?
        - Differentiate packages of support based on levels of disability/ poverty status/ income/ number of prior episodes of TB?
    - For how long should this support be provided?
    - What should be required of the DR-TB patient to receive the support?
    - What should *not* be provided?
    - How can technology be used to support this intervention?
        - Prompt for remote monitoring and drug delivery through shipper apps

Priortization to improve treatment outcomes & catastrophic costs

- Even if this differs from the intervention you proposed, what do you feel is the most important type of support for DR-TB patients to improve DR-TB *treatment outcomes*? Why?
- What do you feel is the most important type of support for DR-TB patients to reduce *catastrophic costs*? Why?

For peer review only

Provision of support

- In your role, do you have capacity to assist with the provision of support to people with DR-TB?
- If this work was to be conducted in the facility where you work, who should provide it?

Is there anything else you would like to tell us?

For peer review only

Focus Group Discussions (Current/Former DR-TB patients)

Experiences on TB treatment

- While on treatment, what types of challenges have you faced?
- What types of support do you need during DR-TB treatment? Why?

Barriers to TB treatment

- From your experience, what is the biggest obstacle that prevents patients from completing treatment?
- What are the main factors that cause catastrophic costs or poverty during DR-TB treatment?

Types of support

- If you could design any type of program to help people through DR-TB treatment, not thinking about the cost, what would that program include?

***If the participants mention a type of support listed below, then ask the questions for that form of support. If they do not bring up a form of support that is outlined below, then prompt them to discuss.*

Social health insurance

- Which types of DR-TB patients should receive health insurance? Why?
- Who should provide SHI to DR-TB patients?
- For how long should SHI be provided to people with DR-TB?
- How effective is SHI at alleviating the costs of DR-TB care?

Education

- On what types of information should DR-TB patients receive counselling?
- When should this information be given during the duration of treatment?
- Thinking back the to the counselling you received, if you could change anything about this counselling, what would you change?

Nutritional support

- What types of food are needed for people with DR-TB?
- What types of supplements are needed during DR-TB treatment?
- Which types of people with DR-TB should be offered nutritional support, if it is offered?

For peer review only

  - Differentiate packages of support based on levels of disability/ poverty status/ income/ number of prior episodes of TB?
- For how long do DR-TB patients need nutritional support?
  - Prompt: during the intensive phase or throughout all of treatment?
- What are the benefits of providing food and/or supplements?
- Do you see any drawbacks to the provision of food and/or supplements?

Cash transfers

- Which types of DR-TB patients should receive the cash transfer? Why?
  - Differentiate cash amounts based on levels of disability/ poverty status/ income/ number of prior episodes of TB?
- For how long should cash be given to people with DR-TB?
- What are the benefits and drawbacks of cash transfers compared to other types of social support?
- How should cash be spent?
- How should cash not be spent?

Transport vouchers

- How do you feel about transportation vouchers being given to people with DR-TB?
- How do transport vouchers address the needs of people with DR-TB?
- Which types of patients should be offered transport vouchers, if they are offered?
  - Differentiate amount of transport voucher based on distance from clinic/ levels of disability/ poverty status/ income/ number of prior episodes of TB?
- How willing do you feel DR-TB patients would be to participate in a project that provided transport vouchers?
- For how long should transport vouchers be given to people with DR-TB?
- What are the benefits of transport vouchers compared to cash payments?

At-home medicine delivery

- How do you feel about medicine being delivered to the homes of people with DR-TB through an app such as Grab, Ahamove, Bee, or another shipper?
- How would medicine delivery address the needs of people with DR-TB?
- Which types of patients should be offered medicine delivery?
- How willing do you feel DR-TB patients would be to receive medicine delivery?
- For how long should medicine be delivered to the homes of people with DR-TB?

For peer review only

Remote monitoring

- How do you feel about attending appointments with your doctor online?
- How would online appointments address the needs of people with DR-TB?
- Which types of patients should be offered remote monitoring or telemedicine?
- How willing do you feel DR-TB patients would be to receive online consultations of in-person meetings?

Patient support groups

- What is your opinion of DR-TB patient support groups?
  - Prompt- in person vs. online support groups
- What types of challenges do patient support groups best address?
- What issues should patient support groups discuss?
- Which types of DR-TB patients would benefit the most from patient support groups?
- What types of benefits would DR-TB patients gain from a support group?
- How willing do you feel people with DR-TB would be to participate patient support groups?
  - Prompt- in person vs. online support groups
- During which stage of DR-TB treatment would a patient support group be most effective to support the patient?
- Who is the most effective type of person to lead the patient support group?
  - Prompt: Peer-led, DTU-led or psychologist-led
- How would you feel about hosting a patient support group?

Access to a psychologist/ psychiatrist for DR-TB patients

- What mental health support services have you required during your treatment?
- What resources were available to you for psychological or psychiatric problems, if any?
- How willing do you feel DR-TB patients would be to meet with a mental health professional during their treatment?
- How would access to a mental health professional improve DR-TB treatment outcomes?

Access to testing and preventative therapy

- How can patient's families best be supported to go for TB testing?
- What barriers do families of people with TB face in accessing TB testing?
- What is the best way to improve LTBI treatment initiation rates among DR-TB contacts?

For peer review only

- What barriers do families of people with DR-TB face in accessing LTBI testing and treatment?

Priortization to improve treatment outcomes & catastrophic costs

- Following this discussion, what do you feel is the most important type of support for DR-TB patients to improve their *treatment outcomes*? Why?
- What do you feel is the most important type of support for TB patients to reduce catastrophic costs? Why?

Is there anything else you would like to tell us?

For peer review only

COREQ (COnsolidated criteria for REporting Qualitative research) Checklist

A checklist of items that should be included in reports of qualitative research. You must report the page number in your manuscript where you consider each of the items listed in this checklist. If you have not included this information, either revise your manuscript accordingly before submitting or note N/A.

Topic	Item No.	Guide Questions/Description	Reported on Page No.
**Domain 1: Research team and reflexivity**			
Personal characteristics			
Interviewer/facilitator	1	Which author/s conducted the interview or focus group?	5
Credentials	2	What were the researcher's credentials? E.g. PhD, MD	5
Occupation	3	What was their occupation at the time of the study?	5
Gender	4	Was the researcher male or female?	5
Experience and training	5	What experience or training did the researcher have?	5
Relationship with participants			
Relationship established	6	Was a relationship established prior to study commencement?	4
Participant knowledge of the interviewer	7	What did the participants know about the researcher? e.g. personal goals, reasons for doing the research	4
Interviewer characteristics	8	What characteristics were reported about the inter viewer/facilitator? e.g. Bias, assumptions, reasons and interests in the research topic	5
**Domain 2: Study design**			
Theoretical framework			
Methodological orientation and Theory	9	What methodological orientation was stated to underpin the study? e.g. grounded theory, discourse analysis, ethnography, phenomenology, content analysis	6
Participant selection			
Sampling	10	How were participants selected? e.g. purposive, convenience, consecutive, snowball	4
Method of approach	11	How were participants approached? e.g. face-to-face, telephone, mail, email	4
Sample size	12	How many participants were in the study?	4
Non-participation	13	How many people refused to participate or dropped out? Reasons?	4
Setting			
Setting of data collection	14	Where was the data collected? e.g. home, clinic, workplace	5
Presence of non-participants	15	Was anyone else present besides the participants and researchers?	4
Description of sample	16	What are the important characteristics of the sample? e.g. demographic data, date	4-5
Data collection			
Interview guide	17	Were questions, prompts, guides provided by the authors? Was it pilot tested?	5
Repeat interviews	18	Were repeat inter views carried out? If yes, how many?	5
Audio/visual recording	19	Did the research use audio or visual recording to collect the data?	5
Field notes	20	Were field notes made during and/or after the inter view or focus group?	5
Duration	21	What was the duration of the inter views or focus group?	5
Data saturation	22	Was data saturation discussed?	4
Transcripts returned	23	Were transcripts returned to participants for comment and/or	5

For peer review only

Topic	Item No.	Guide Questions/Description	Reported on Page No.
		correction?	
**Domain 3: analysis and findings**			
Data analysis			
Number of data coders	24	How many data coders coded the data?	6
Description of the coding tree	25	Did authors provide a description of the coding tree?	6
Derivation of themes	26	Were themes identified in advance or derived from the data?	6
Software	27	What software, if applicable, was used to manage the data?	6
Participant checking	28	Did participants provide feedback on the findings?	6
Reporting			
Quotations presented	29	Were participant quotations presented to illustrate the themes/findings? Was each quotation identified? e.g. participant number	8-11
Data and findings consistent	30	Was there consistency between the data presented and the findings?	8-11
Clarity of major themes	31	Were major themes clearly presented in the findings?	8-11
Clarity of minor themes	32	Is there a description of diverse cases or discussion of minor themes?	8-11

Developed from: Tong A, Sainsbury P, Craig J. Consolidated criteria for reporting qualitative research (COREQ): a 32-item checklist for interviews and focus groups. *International Journal for Quality in Health Care*. 2007. Volume 19, Number 6: pp. 349 – 357

Once you have completed this checklist, please save a copy and upload it as part of your submission. DO NOT include this checklist as part of the main manuscript document. It must be uploaded as a separate file.

For peer review only

BMJ Open

What matters most? A qualitative study exploring priorities for supportive interventions for people with tuberculosis in urban Viet Nam

Journal:	*BMJ Open*
Manuscript ID	bmjopen-2023-076076.R1
Article Type:	Original research
Date Submitted by the Author:	07-Aug-2023
Complete List of Authors:	Smith, Isabel; Karolinska Institute, WHO Collaborating Centre for Social Medicine and Tuberculosis, Department of Global Public Health Sciences<br>Forse, Rachel; Karolinska Institutet, WHO Collaborating Centre for Social Medicine and Tuberculosis, Department of Global Public Health Sciences; Friends for International TB Relief, TB Programs<br>Sidney, Kristi; Karolinska Institutet, WHO Collaborating Centre for Social Medicine and Tuberculosis, Department of Global Public Health Sciences<br>Thanh, Nguyễn Thị; Centre for Development of Community Health Initiatives<br>Nguyen, Lan; IRD VN<br>Phan, Thi Hoang Yen; Centre for Development of Community Health Initiatives<br>Nguyen, Han; Friends for International Tuberculosis Relief<br>Codlin, Andrew; Friends for International Tuberculosis Relief; Karolinska Institute, WHO Collaborating Centre for Social Medicine and Tuberculosis, Department of Global Public Health Sciences<br>Vo, Luan ; Friends for International TB Relief,<br>Nguyen, Nga Thi Thuy; Friends for International Tuberculosis Relief<br>Khan, Amera; Stop TB Partnership<br>Creswell, Jacob; Stop TB Partnership,<br>Pham Huy, Minh; USAID Vietnam<br>Basu, Lopa; USAID Vietnam<br>Lönnroth, Knut; Karolinska Institute, WHO Collaborating Centre for Social Medicine and Tuberculosis, Department of Global Public Health Sciences<br>Nguyen, Binh Hoa; Vietnam National Tuberculosis Program<br>Nguyen, Viet Nhung; National Lung Hospital , National TB Program<br>Atkins, Salla; Tampere University, New Social Research and Faculty of Social Sciences; Karolinska Institute, WHO Collaborating Centre for Social Medicine and Tuberculosis, Department of Global Public Health Sciences
**Primary Subject Heading**:	Global health
Secondary Subject Heading:	Evidence based practice, Health policy, Health services research, Public health, Qualitative research
Keywords:	Tuberculosis < INFECTIOUS DISEASES, Health policy < HEALTH SERVICES ADMINISTRATION & MANAGEMENT, Public health <

	INFECTIOUS DISEASES

SCHOLARONE™
Manuscripts

I, the Submitting Author has the right to grant and does grant on behalf of all authors of the Work (as defined in the below author licence), an exclusive licence and/or a non-exclusive licence for contributions from authors who are: i) UK Crown employees; ii) where BMJ has agreed a CC-BY licence shall apply, and/or iii) in accordance with the terms applicable for US Federal Government officers or employees acting as part of their official duties; on a worldwide, perpetual, irrevocable, royalty-free basis to BMJ Publishing Group Ltd ("BMJ") its licensees and where the relevant Journal is co-owned by BMJ to the co-owners of the Journal, to publish the Work in this journal and any other BMJ products and to exploit all rights, as set out in our licence.

The Submitting Author accepts and understands that any supply made under these terms is made by BMJ to the Submitting Author unless you are acting as an employee on behalf of your employer or a postgraduate student of an affiliated institution which is paying any applicable article publishing charge ("APC") for Open Access articles. Where the Submitting Author wishes to make the Work available on an Open Access basis (and intends to pay the relevant APC), the terms of reuse of such Open Access shall be governed by a Creative Commons licence – details of these licences and which Creative Commons licence will apply to this Work are set out in our licence referred to above.

Other than as permitted in any relevant BMJ Author's Self Archiving Policies, I confirm this Work has not been accepted for publication elsewhere, is not being considered for publication elsewhere and does not duplicate material already published. I confirm all authors consent to publication of this Work and authorise the granting of this licence.

For peer review only

What matters most? A qualitative study exploring priorities for supportive interventions for people with tuberculosis in urban Viet Nam

Authors

Isabel Smith[1], Rachel Forse[1,2], Kristi Sidney-Annerstedt[1], Thanh Thi Nguyen[3], Lan Nguyen[4], Thi Hoang Yen Phan[3], Han Nguyen[2], Andrew J. Codlin[1,2], Luan Nguyen Quang Vo[2], Nga Nguyen[2], Amera Khan[5], Jacob Creswell[5], Minh Pham[6], Lopa Basu[6], Knut Lönnroth[1], Hoa Binh Nguyen[7], Nhung Viet Nguyen[7], Salla Atkins[1,8]

1. WHO Collaborating Centre for Social Medicine and Tuberculosis, Department of Global Public Health, Karolinska Institute, Stockholm, Sweden
2. Friends for International TB Relief (FIT), 1/21 Le Van Luong, Nhan Chinh, Thanh, Xuan, Ha Noi, Viet Nam
3. Centre for Development of Community Health Initiatives (CHI), 1/21 Le Van Luong, Nhan Chinh, Thanh Xuan, Ha Noi, Viet Nam
4. IRD VN, 68B Nguyen Van Troi, Ward 3, Phu Nhuan, Ho Chi Minh City, Viet Nam
5. Stop TB Partnership, Chem. du Pommier 40, 1218 Le Grand-Saconnex, Switzerland
6. United States Agency for International Development (USAID), 15/F, Tung Shing Building, 2 Ngo Quyen, Ha Noi, Viet Nam
7. National Lung Hospital/National TB Control Programme, 463 Hoang Hoa Tham, Vinh Phu, Ba Dinh, Ha Noi, Viet Nam
8. Faculty of Social Sciences, Tampere University, Arvo Ylpön katu 34, Tampere, Finland

Corresponding Author:
Rachel Forse
rachel.forse@tbhelp.org

Abstract

Introduction: The health and economic burden of Tuberculosis (TB) in urban Viet Nam is high. Social protection and support interventions can improve treatment outcomes and reduce costs. However, evidence regarding optimal strategies in this context is lacking. This study aimed to increase understanding of what people with TB and healthcare providers (HCPs) perceive as important to improve TB treatment outcomes and reduce costs.

Methods: We conducted qualitative focus group discussions (7 groups, n=30) and key informant interviews (n=4) with people with drug-susceptible and multidrug-resistant TB and HCPs in Ha Noi and Ho Chi Minh City. Topic guides covered perspectives on and prioritization of different forms of social protection and support. Data were analysed using reflexive thematic analysis and interpreted using a Framework for Transformative Social Protection.

Results: We identified three themes and seven sub-themes. The first theme, 'Existing financial safety nets are essential, but could go further to support people affected by TB', highlights that support to meet the medical costs of TB treatment and flexible cash transfers are a priority for people with TB and HCPs. The second, 'It is important to promote "physical and spiritual health" during TB treatment', demonstrates that extended psychosocial and nutritional support would encourage people with TB during their treatment. The third, 'Accessibility and acceptability are critical in designing social support interventions for

For peer review only - http://bmjopen.bmj.com/site/about/guidelines.xhtml

people with TB', shows the importance of ensuring that support is accessible and proportional to the needs of people with TB and their families.

Conclusions: Accessible interventions that incorporate financial risk protection, nutritional and psycho-social support matter most to people with TB and HCPs in urban Viet Nam to improve their treatment outcomes and reduce catastrophic costs. This study can inform the design of stronger person-centred interventions to advance progress towards the goals of the World Health Organization's End TB Strategy.

Strengths and limitations of this study

- This study included a diverse group of participants, enhancing the trustworthiness and validity of the findings, and ensuring that the resulting recommendations took the perspectives of both people with TB and healthcare providers into account. This is particularly important given the role of healthcare providers in delivering and connecting people with TB to available support.
- Interviews were conducted in a neutral space rather than in a health facility to mitigate social desirability bias.
- This study was conducted solely in urban settings in Viet Nam, therefore the findings may not be generalisable to rural settings within Viet Nam or to other high burden countries.
- The majority of the interviews were conducted during COVID-19 and it is possible that the needs of people with TB may have shifted since data collection.
- The analysis was conducted on English translations of the interviews. This may have influenced the interpretation of data; however, the study took a rigorous, collaborative approach to limit potential effects of ethnocentrism on the analysis.

Introduction

Tuberculosis (TB) caused 1.6 million deaths in 2021 (1). It disproportionately affects poorer populations who are more vulnerable to TB infection, development of active TB disease, and worse treatment outcomes (1–4). Further, TB can push affected households into poverty (5,6) through the accrual of direct and indirect costs. These can amount to catastrophic costs, defined by the WHO as total costs due to TB exceeding 20% of annual household income (1). The impoverishing effects of TB may have been compounded by COVID-19, particularly for more vulnerable populations (7). Eliminating TB will require both biomedical innovation and socio-economic interventions, as outlined in the World Health Organization's (WHO) End TB Strategy (8).

The provision of social protection schemes must be a part of the response to end TB. There is consistent evidence that spending on broad social protection, including population-level poverty reduction schemes such as pensions and child support, is inversely associated with TB incidence and mortality (9). Some estimates indicate that expansion of social protection initiatives could reduce global TB incidence by up to 76% (10). Furthermore, social protection schemes for people with TB such as cash transfers, transport vouchers and nutritional support have been shown to defray out-of-pocket costs, improve treatment outcomes or improve treatment adherence in various high-burden settings (11, 12, 13). For example, cash transfers for people with TB in Peru led to improved treatment success and

For peer review only - http://bmjopen.bmj.com/site/about/guidelines.xhtml

decreased likelihood of incurring catastrophic costs (14, 15). While most social protection interventions for TB-affected household focus on financial or nutritional support (16), social and psychological support interventions have also contributed to improved treatment outcomes and reduced costs (14, 17).

Viet Nam is one of the world's highest burden countries for TB and multidrug-resistant TB (MDR-TB) (18), and TB incidence in Viet Nam is particularly high in urban areas (19). Despite the provision of free diagnostics and anti-TB treatment by the National TB Program (NTP), high coverage of social health insurance (SHI) covering medical costs (20) and subsidies from the Global Fund for people with DR-TB (19, 21), a 2016 patient cost survey found that 63% of households containing a person with drug-susceptible (DS-TB) and 98% of households containing a person with MDR-TB experienced catastrophic costs due to the disease (22). The government of Viet Nam is aiming to achieve 100% coverage of SHI by 2025 (20) and has announced that in mid-2022, TB care at district-level facilities will be included under the national SHI system (23). While the full implications of this change are unclear, this likely will increase user fees and out-of-pocket payments for the majority of individuals undergoing DS-TB care, further highlighting the importance of social protection measures for people with TB.

Viet Nam's NTP has identified the impoverishing effects of TB as a priority for action (24) and has established a national fund (Patients Support to Fight TB) to support poor and uninsured people with TB (for example, by purchasing SHI on their behalf) (22,24). However, there is limited context-specific understanding of the optimal ways in which supportive interventions can improve treatment outcomes and reduce costs for people with TB in urban Viet Nam. A prior study exploring the acceptability of cash transfers and SHI among people with TB, healthcare providers (HCPs), policymakers and members of the public in Viet Nam (25) indicated that further research into preferences for supportive interventions was required. Therefore, this study aims to increase understanding of what people with TB and HCPs in urban Viet Nam perceive as important in the design of interventions to reduce costs and improve TB treatment outcomes.

Methods

Study design

We conducted an exploratory qualitative study using semi-structured focus group discussions (FGDs) and key informant interviews to understand the perceptions of people with TB and TB HCPs in urban Viet Nam regarding supportive interventions to improve treatment outcomes and reduce costs. The Consolidated Criteria for Reporting Qualitative Research checklist (26) was used to ensure comprehensive reporting.

Study setting

The study was conducted in two cities in Viet Nam, Ha Noi and Ho Chi Minh City, where a consortium of local NGO partners works with the NTP to deliver enhanced TB care and conduct operational research (27,28). Data were collected in July-September 2020 and February 2022 in the context of COVID-19.

At the time of the study, the NTP provided anti-TB drugs and consultations free-of-charge in the public sector. SHI could not be used to subsidise first-line TB medications (22) at district-

For peer review only - http://bmjopen.bmj.com/site/about/guidelines.xhtml

level facilities but covered 80% of eligible medical expenditure for the majority of people (21). To obtain SHI, people must register and pay an annual fee, typically for their entire household (29). For most people, SHI is registered at the commune or district level and is not valid at provincial or national hospitals without a referral (30). People with TB meet other costs of treatment through out-of-pocket payments and social protection schemes supported by government programs, the Global Fund and other funding sources (22).

In 2021, the TB incidence rate in Viet Nam was 173 cases per 100,000 people and there were over 2,600 new cases of MDR-TB (1). Among those treated, treatment was successful in 91% of new and relapse cases and in 74% of MDR-TB cases (1).

Participant selection

Purposive sampling was used to select participants with a range of experiences of TB treatment who had not previously engaged in the NGOs' services. TTN contacted District TB Units (DTUs) in Ha Noi and Ho Chi Minh City who provided lists of potential participants: people who had been successfully or unsuccessfully[1] treated for bacteriologically-confirmed drug-susceptible (DS-) TB, people being treated for bacteriologically-confirmed MDR-TB with a 9- or 20-month regimen, and TB HCPs employed at national, provincial and district levels.

An assessment of information power (31) concluded that a moderate sample size was required to address the study's aim. The sample size was guided by estimates based on previous studies and data saturation was discussed following each FGD or interview. A total of 66 individuals were invited, but 33 (13 men, 20 women) declined to participate, mostly due to a lack of interest or distance from the interview site. One participant was accompanied by their caregiver, who also gave consent to participate. Demographic characteristics are reported in Table 1.

Table 1: Participant demographic characteristics.

	Sex (n)		Total (n)
	**Female**	**Male**	
All Participants	12¶	21	33¶
Location			
Ha Noi	10¶	12	22¶
Ho Chi Minh City	2	9	11
Participant Group			
People successfully treated for DS-TB	1¶	7	8¶
People unsuccessfully treated for DS-TB	0	6	6
People with MDR-TB	4	6	10
District TB Health Care Providers (HCPs)	3	2	5
National/Provincial TB HCPs	3	1	4

Note: ¶ Indicates that at least one participant was accompanied by their caregiver.

Data collection

[1] Unsuccessfully treated defined as retreated or relapse cases.

For peer review only - http://bmjopen.bmj.com/site/about/guidelines.xhtml

For peer review only

Data were collected by female NGO staff trained and experienced in conducting qualitative research, and was directly overseen by TTN, a qualitative researcher and NGO project manager with five years of qualitative research experience. All participants were informed about the purpose of the study when they were invited to participate by telephone, although not provided with details about the interviewers' motivations or biases. They provided written informed consent prior to data collection. Participants were provided with refreshments and 300,000 VND ($12.73 USD, 23,571.8 VND: 1 USD, xe.com) to cover travel costs.

Four FGDs with people with DS-TB and one FGD with DTU HCPs were conducted in July-September 2020 in dedicated offices. Two FGDs with people with MDR-TB and four interviews with National/Provincial TB HCPs were conducted in February 2022 at the Ha Noi Lung Hospital, the NGO offices or over Zoom. FGDs were conducted in person and lasted 91-188 minutes. Key informant interviews were conducted via Zoom and lasted 46-90 minutes. The study aimed to recruit five participants per FGD, however some were smaller due to enrolment challenges and may be better classified as group interviews. No repeat interviews were conducted. Further details can be found in Appendix A.

Topic guides were developed iteratively by TTN, RF and KSA and included questions on challenges during TB treatment and different forms of support; complete topic guides are in Appendix B. Questions on digital support were added to later FGDs and interviews due to its increased use during the COVID-19 pandemic. Participants were also asked to rank interventions that they thought were most important for improving treatment outcomes and reducing costs.

FGDs were conducted in Vietnamese, audio recordings were transcribed by NGO staff and then translated into English by an external service provider in Viet Nam. The translations were checked by bilingual study staff. Field notes were recorded following the FGDs and KIIs. Participant data were pseudonymised, stored securely and not shared beyond the study team. Transcripts were not shared with participants for comment.

Patient and Public Involvement
People with TB, HCPs, policymakers and members of the public were involved in this research through the development of topic guides. Their responses to a prior qualitative interview study on the acceptability of cash and SHI (25) informed the development of a list of forms of social protection to explore further in this study. The results of this research were disseminated to government officials, the NTP and patient communities in roundtable discussions.

Data analysis
Data were analysed using reflexive thematic analysis (32, 33). IS, a global health master's student, developed the coding framework with SA, an experienced qualitative researcher, and coded the data using 'Dedoose' software (version 9.0.54) (34) before identifying themes based on recurrent patterns. The analysis was conducted through an iterative process. Codes, categories, themes and sub-themes were verified by SA, KSA and RF. An example is illustrated in Table 2.

For peer review only - http://bmjopen.bmj.com/site/about/guidelines.xhtml

Table 2: Examples of quotes with corresponding code descriptions, codes, themes and sub-themes.

Example quote	Code description	Code	Sub-theme	Theme
"There are many aspects that make people give up including travel expenses. So, thanks to this voucher support during the treatment a lot of sick people would benefit."	The costs of TB are a barrier to completing treatment	Financial problems affect adherence	Enabling people with TB to meet medical costs is a priority for support	Existing financial safety nets are essential, but could go further to support people with TB
"When I was sad, my family encouraged me."	People with TB are encouraged and supported by friends and family	Emotional support from friends and family	Psychosocial support is fundamental for people with TB to "feel secure in treatment"	It is important to promote "physical and spiritual health" during TB treatment
"Besides, during the time of taking this medicine, it was very tiring and exhausting, so I had to drink a lot of water, take tonics during that course."	TB drugs can make people with TB feel tired and weak	Treatment makes you tired and weak	Nutritional supplementation is viewed as important for people with TB to be able to "fight against the disease"	It is important to promote "physical and spiritual health" during TB treatment
"When they know it, honestly, they are afraid to examine and test. If they are ignored about how it is dangerous, they shall still omit it."	Contacts of people with TB don't want to get tested	Contacts reluctant to test	Increasing access to TB diagnostic and preventive services is an important part of social protection	Accessibility and acceptability are critical in designing social support interventions for people with TB

Themes were interpreted in line with the Framework for Transformative Social Protection which defines social protection as a range of policies or initiatives which help vulnerable people to move sustainably out of poverty by acting on their financial risk and enhancing their social status (35).

Results

We generated three themes and five sub-themes from the data, summarised in Table 3 and discussed below. Participants also spoke about challenges experienced during TB treatment which are covered extensively elsewhere. We have indicated where HCPs and people with TB differed in opinion, and used 'participants' where they concurred.

Table 3: Themes and sub-themes identified during reflexive thematic analysis.

Themes		Sub-themes	
1	Existing financial safety nets are essential but could go further to support people with TB	1.1	Enabling people with TB to meet medical costs is a priority for support
		1.2	Flexible financial support can give people with TB more agency

For peer review only - http://bmjopen.bmj.com/site/about/guidelines.xhtml

2	It is important to promote "physical and spiritual health" during TB treatment	2.1	Psychosocial support is fundamental for people with TB to "feel secure in treatment"
		2.2	Nutritional supplementation is important for people with TB to be able to "fight against the disease"
3	Accessibility and acceptability are critical in designing social support interventions for people with TB	3.1	Support for people with TB can be prioritised by need – there is no one-size-fits-all solution
		3.2	Support should alleviate the burden of people with TB, not add to it
		3.3	Increasing access to TB diagnostic and preventive services is an important part of social protection

Theme 1: Existing financial safety nets are essential but could go further to support people with TB

Sub-theme 1.1: Enabling people with TB to meet medical costs is a priority for support

Participants were concerned about the financial burden of TB and viewed financial support as essential to reducing costs and ensuring good treatment outcomes. Most participants, particularly HCPs, thought that people with TB should not pay any medical costs during their treatment and noted this as their top priority for reducing costs and improving treatment outcomes. Participants valued provision of treatment by the NTP, but many people with TB reported paying different amounts depending on treatment location, treatment regimen or insurance status.

Participants described SHI as important, but difficult to access. Many people with TB reported that SHI registration was slow and expensive (particularly for households) and many were not registered at the right level. In fact, people with TB and HCPs alike reported that seeking care outside of the primary registered health facility was challenging. HCPs also noted difficulties with making claims, calculating payments and deductibles.

However, many participants prioritised SHI for reducing the costs of TB care. One male District TB Unit HCP summarised this view, saying *"We should focus on basic support. For example, health insurance is necessary, then testing".* Overall, participants thought that uninsured people with TB should be provided with SHI, that SHI coverage or the NTP's provision should be expanded to cover more costs including diagnostic and confirmatory testing (including X-rays and GeneXpert), hospitalisation fees and supplements and that registration should be made simpler.

Sub-theme 1.2: Flexible financial support can give people with TB more agency

Many participants thought that cash transfers were important for mitigating out-of-pocket expenses and income loss and improving treatment outcomes. For people with TB in particular, cash transfers were a priority because they saw them as simple, quick and versatile, and could be used to solve upfront costs such as hospitalisation fees. One male participant who was successfully treated for DS-TB expressed that *"Cash, in general. Cash solves many things. It's simple"*. Further, cash transfers were seen by most as easier to

For peer review only - http://bmjopen.bmj.com/site/about/guidelines.xhtml

implement than vouchers, easier to access than loans, and could give people with TB more independence to spend at their discretion. However, other people with TB spoke about a potential to misuse cash transfers (e.g., purchasing tobacco) and therefore thought that transport or nutrition vouchers were a better way to provide financial support.

People with TB also wanted support to find suitable employment to counter lost income. They indicated that career counselling, help with finding a new, less physically demanding job through connection with a job centre or job placement, or lending money to invest in their career would be beneficial. This was seen as more useful than vocational support, with one male participant who was successfully treated for DS-TB saying "*After training, there must be available jobs, or else, it's just a waste of time, it's very difficult to re-train*".

Theme 2: It is important to promote "physical and spiritual health" during TB treatment

Sub-theme 2.1: Psychosocial support is key for people with TB to "feel secure in treatment"
People with TB thought that financial support could also reduce worry and provide a sense of security during treatment, and thus improve mental health. This was summarised by one participant who was unsuccessfully treated for DS-TB who said that *"Sometimes, it is only a small part of a support package, but it provides them [the beneficiary] with something for their spirit- to help them make their best effort in their treatment. They feel better and cared for by society".*

All participants spoke about the negative impact that TB has on mental health. One female National/Provincial TB HCP described it as: *"There are patients who know they are depressed, bored, feel that they are not getting better, they are still tired from taking medication, the illness, they want to give up".* More broadly, people with TB frequently referred to how pessimism can make it harder to recover and about the importance of feeling secure in their treatment.

As a result, many participants recognised a need for psychological, emotional and spiritual support to promote better treatment outcomes. People with TB described emotional support from friends and relatives as essential, but recognised a need for increased access to a psychologist or psychiatrist, particularly for people with MDR-TB. Similarly, TB HCPs identified a need for psychological training given the scarcity of mental health specialists, as highlighted by this female National/Provincial TB HCP *"Psychiatrists are very, very rare. Even our [national-level] hospital does not have any. So sometimes, we see our patients and sincerely share from our hearts and our thoughts. But in terms of psychological-expertise, we have no specialization in psychology"*. However, some people with DS-TB reported no psychological problems and were reluctant to engage with proposed support.

Participants also thought that patient support groups could help people with TB to learn from one another and encourage optimism about treatment. This was summarised by one female participant with MDR-TB as *"When we undergo the treatment, we talk to people who don't have the disease, they won't understand us… But it's easier to talk to people who feel the same way as we do"*. Participants thought that these groups should be led by experts, whether that be HCPs, mental health specialists, or others with TB.

For many participants, particularly people with MDR-TB, comprehensive TB education and guidance from HCPs about TB treatment was an important part of feeling secure in their

For peer review only - http://bmjopen.bmj.com/site/about/guidelines.xhtml

treatment, for example by helping them to manage side effects. People with TB thought that information should be provided by doctors at the start of treatment and available throughout. One participant with MDR-TB suggested that specialist pharmacists would be able to answer questions about side-effects of medicines. HCPs also suggested using leaflets, recorded messages and public media to share information about TB prevention and treatment.

Sub-theme 2.2: Nutritional supplementation is viewed as important for people with TB to be able to "fight against the disease"

Most participants thought that good nutrition and rest were essential to recovery and a priority for support, particularly for people with MDR-TB. One HCP at a District TB Unit summarised that *"tuberculosis is exhausting. If you are malnourished, you need energy, fibre, and vitamins".* Some participants thought that people with TB should be supported directly with food but many considered cash to be more helpful.

Participants also thought that supplements and tonics were essential for controlling side effects (e.g., through detoxifying the liver). All participants thought that they should be included in a support package rather than paid out-of-pocket. One National/Provincial TB HCP highlighted how costs can accumulate throughout treatment by explaining that *"Some patients don't have money to buy them, so they just receive the TB medications, no supplements. Then they have liver and kidney issues and get hospitalised. Then, the cost of liver and kidney detox would be many times higher than the cost of liver supplements they should have paid for in the beginning".*

Theme 3: Accessibility and acceptability are critical in designing social support interventions for people with TB

Sub-theme 3.1: Support for people with TB can be prioritised by need – there is no one-size-fits-all solution

Participants were asked who should be prioritised to receive each type of support and when it should be delivered. Some participants in each group thought that all people with TB should be supported equally, reasoning that prioritisation would cause jealousy, and that it should last throughout treatment. However, most participants thought that support for people with TB should be tailored to their individual needs.

Participants thought that people struggling financially or with more severe disease had greater needs for support and should therefore be prioritised. For example, one male participant successfully treated for DS-TB suggested that *"TB patients should be all supported, dividing by severity or circumstances, the poor or persons living in difficult situations, second treatment or drug resistance."*. Similarly, when prompted to prioritise when support was given, many people with TB suggested it be given during the intensive phase of treatment. On the other hand, many National/Provincial TB HCPs were explicit that support should be highly specific, with transport vouchers according to distance travelled or financial support according to days of hospitalisation.

For peer review only - http://bmjopen.bmj.com/site/about/guidelines.xhtml

Sub-theme 3.2: Support should alleviate the burden of people with TB, not add to it
Speaking from past experiences, TB HCPs raised concerns that defining eligibility criteria for support would create overly burdensome registration systems that impede access. One District TB HCP summarised that *"If you offer, but in the end you can't support due to the inflexible procedures... You support nobody."*

People with TB were also concerned with the practicality of proposed support options. Transport vouchers were viewed positively because they help people attend appointments and adhere to treatment. However, delivering food was viewed as a waste of time and resources. Similarly, people with TB viewed vocational training as impractical with one male participant who was successfully treated for DS-TB saying *"As old people have a certain job, it's hard to change to another. It's okay to just introduce an easier job for them, vocational training is not feasible."*

Additionally, participants wanted support to be convenient and accessible. They viewed current support as insufficient or inaccessible due to inflexible procedures or inadequate publicity. People with DS-TB viewed patient support groups positively but were reluctant to participate due to the time commitment and fears over re-infection. District TB HCPs had concerns about adding to their own overburdened workload by running these groups in person. Participants thought that online support groups would increase access to support in the context of limited staff resources and suggested using online forums on social media platforms to ask questions and share information. However, they had concerns about the accessibility of online support for older people.

Similarly, people with MDR-TB and National/Provincial TB HCPs viewed remote delivery of medication and online consultations as an additional way to access support when necessary, such as during periods of intense COVID-19 restrictions but people with TB did not want to replace in-person appointments entirely.

Sub-theme 3.3: Increasing access to TB diagnostic and preventive services is an important part of social protection
Many participants thought that any support given should extend beyond the individual. Access to testing and preventive treatment for families affected by TB was prioritised by many people with TB and National/Provincial TB HCPs as an important way to reduce onwards transmission and reduce costs. People with TB suggested that their families be provided with home testing kits or transport vouchers to enable testing without incurring costs. One individual with MDR-TB highlighted the potential socio-protective effects of doing so, saying *"It's good for my family to go to the doctor to get screened... now there's a support system, it'll help with the expenses... If they go for screening, the disease is detected early, good for them. If you don't do it until it's severe, treatment will be more difficult."*.

Discussion

This study demonstrates that people with DS- and MDR-TB and TB HCPs perceive mixed interventions that include social support and social protection measures to be important for reducing costs and improving TB treatment outcomes. In particular, measures that 'matter most' include provision of SHI and flexible cash transfers to meet the costs of treatment,

For peer review only - http://bmjopen.bmj.com/site/about/guidelines.xhtml

nutritional support that enables people with TB to manage their side-effects, and psychosocial support that encourages them during treatment. Recommendations for the design of supportive interventions, as informed by our findings regarding participants' priorities, are summarised in Table 4.

Table 4: Recommendations for action based on participant responses.

Theme	Recommendation
Existing financial safety nets are essential but could go further to support people with TB	• Ensure all people with TB are covered by Viet Nam's national Social Health Insurance scheme, and plug gaps in coverage for those who are not<br>• Provide cash transfers to people with TB to be spent at their discretion<br>• Establish, or link people with TB to existing, career counselling services or job placements
It is important to promote "physical and spiritual health" during TB treatment	• Increase availability of trained psychologists and psychiatrists where possible, or provide psychological training for TB HCPs, particularly for people with multi-drug resistant TB (MDR-TB)<br>• Establish online peer support forums using platforms such as messenger applications (e.g., WhatsApp or Zalo) involving people with Tuberculosis (TB) and TB Healthcare Practitioners (HCPs) to act as an information-sharing platform and enable patients to report and receive assistance for side-effects<br>• Ensure comprehensive TB education to help people with TB manage their treatment and reduce worry, including guided counselling sessions between people with TB and TB doctors at the beginning of treatment as standard of care
Accessibility and acceptability are critical in designing social support interventions for people with TB	• Continue and extend existing transport and nutritional support, particularly for people with MDR-TB<br>• Increase access to testing and treatment for TB infection and ensure all contacts of people with TB are tested for TB<br>• Differentiate all support according to multidimensional need, considering economic status and severity of disease as appropriate

To our knowledge, this is the first study to explore how people directly affected by TB prioritise a broad range of different social protection and psychosocial support interventions. This study adds to existing evidence by showing that both people with TB and TB HCPs prioritise interventions that extend beyond SHI and cash transfers, which are currently available in urban Viet Nam and are most common in social protection interventions globally (16). Furthermore, it highlights the broader benefits of social protection, demonstrating how the provision of any support can make people with TB feel more secure in their treatment, as well as mitigating its costs.

For peer review only

These findings can be considered in keeping with a framework for transformative social protection (Figure 1) (35), which extends the definition of social protection beyond financial risk protection to include a broader collection of promotive, protective and transformative interventions that provide both 'safety nets' and 'springboards' out of poverty.

Universal health coverage, including access to health insurance, is a key part of the WHO's End TB Strategy. In this study, many HCPs prioritised SHI over other interventions and suggested that SHI, or existing subsidies, should be extended to cover all treatment costs for all people with TB. Participants also recognised challenges with its implementation, reflecting recent findings in this setting (36), and that catastrophic costs remain high despite high SHI coverage due to non-medical costs and lost income, in line with previous studies (2,22). Therefore, and as recognised by the NTP (24), supportive interventions for people with TB in urban Viet Nam should extend beyond SHI.

There is mounting evidence for the positive impact of cash transfers on reducing costs and improving treatment outcomes for people with TB (14, 15, 37). Therefore, it is perhaps unsurprising that all participant groups in this study identified cash transfers as a priority for both improving outcomes and reducing costs. Furthermore, our study highlighted that cash transfers give people with TB more agency and are simple to manage, as well as being more cost-effective (38). Cash transfers are already provided for poor or uninsured people with TB by Viet Nam's government and for people with DS-TB in certain regions of Ha Noi and Ho Chi Minh City under donor projects (39), so expanding to more people with TB may bring wider benefits.

However, there is an ongoing debate regarding whether cash, vouchers or in-kind support are more effective (38). Targeted provision measures, such as transport or nutrition vouchers, can provide a 'safety net' to avoid the exacerbation of catastrophic costs. Our study demonstrates that alleviating transport costs is also important for people with TB both for improving treatment outcomes, complementing previous findings that transport vouchers can improve treatment adherence and success rates (40, 41). Participants also viewed nutritional and supplement support as key to managing side effects and associated costs, reiterating previous findings (42). However, the evidence for the clinical benefit of nutritional supplementation (43) or of traditional supplements (44) is limited. Consequently, cash transfers and transport vouchers may be a better way to address non-medical costs, with further research required around the benefits of nutritional and traditional supplements.

Promotive interventions that support people with TB to establish a sustainable livelihood can provide a 'springboard' out of poverty (3,35) by targeting the high burden of lost productivity during TB treatment (6, 22, 45).as well as contributing to improved TB treatment outcomes (3). In this study we found that job placements or career counselling were preferred over vocational training and could be an effective way to support people with TB, particularly breadwinners and younger people, to re-enter the workforce. However, there must be opportunities for employment available after such interventions. Given the adverse impact of COVID-19 on employment in urban Viet Nam (46), enabling employment for people with TB may be more important than ever.

For peer review only - http://bmjopen.bmj.com/site/about/guidelines.xhtml

Social protection is one part of a broader set of efforts needed to contain TB (8). Participants in our study also emphasised the importance of social support. In particular, they noted the importance of increasing access to psychological support given the distress experienced during TB treatment (17,47,48). Integrating psychological support into existing care by training TB HCPs, as suggested by our participants, could improve access and has been shown to be effective in another context (49). Informal online patient support groups could also promote wider access to psychosocial support and TB education by allowing people with TB to learn from one another. These may become more acceptable following the COVID-19 pandemic, noting potential difficulties for older people. In line with the framework for transformative social protection, which emphasises both financial and social aspects of vulnerability (35), there is evidence that combined psychological and financial interventions are more effective at improving treatment outcomes than financial support alone (17,50). This could therefore improve supportive interventions for people with TB in urban Viet Nam.

Our participants highlighted the importance of accessibility of TB services and support both for people with TB and their families. This is in line with a recent scoping review which identified a lack of awareness and logistical complexities as key barriers to improving equity through social protection (16). One way in which this could be achieved is through active case finding (ACF) to identify unidentified persons with TB. This is especially important in Viet Nam given that just 46% of estimated new TB cases in 2021 were notified to the government (1). Viet Nam's NTP has recognised the importance of ACF as it can reduce transmission and improve health outcomes over time. Furthermore, there is evidence from Ho Chi Minh City that it can reduce the incurrence of catastrophic costs and can reach more marginalised individuals, increasing equity in TB care (51). ACF could therefore be considered a key part of a wider package of social protection measures in this context and contribute to achieving the End TB goals (7).

Finally, our study suggests that supportive interventions for people with TB could be prioritised to make effective use of resources by allocating based on individual need, taking the agency of people with TB in identifying these needs into account, and targeting the intensive phase of treatment when costs are highest. Differentiated service delivery, in which services are tailored to individual needs, is already a key aspect of person-centred HIV care that has been shown to improve access to and uptake of testing and treatment (52). A similar approach could be taken to allocating packages of support for people with TB, learning from experiences with HIV to ensure that any eligibility criteria did not create barriers to accessing support. However, modes of differentiating support are highly context dependent (40, 52) and further research is required to identify optimal approaches for people with TB in Viet Nam.

Strengths and limitations

A key strength of this study is the inclusion of a diverse group of participants, enhancing trustworthiness and validity and ensuring that the resulting recommendations take their views into account and can therefore inform person-centred and context appropriate intervention design. Some argue that conformity pressures can lead to "groupthink' in FGDs (53). However, participants expressed some different opinions on the best way to support

For peer review only - http://bmjopen.bmj.com/site/about/guidelines.xhtml

people with TB with the costs of treatment and there was broad consensus between participants in different FGDs, suggesting robustness of the findings. Interviews were conducted in the NGO offices rather than in the health facility to mitigate social desirability bias.

A limitation is that this study was conducted solely in urban settings in Viet Nam, therefore the findings may not be generalisable to rural settings within Viet Nam or to other high burden countries. The needs of people with TB may have shifted since data collection given the widespread impact of COVID-19 on health service utilisation and economic wellbeing (54, 55) as well as changes to financing for TB (23).

Further, the analysis was conducted on English translations of the transcripts rather than in Vietnamese, the original language of data-collection, which may have influenced their interpretation (56). However, the study team took a rigorous approach to transcription and translation and there was close collaboration between Vietnamese- and non-Vietnamese-speaking team members to limit the effect of ethnocentrism on the analysis.

Implications and conclusions

Our study highlights that a broad range of social protection and social support measures are required to meet the needs of people with TB in urban Viet Nam and identifies interventions that are important to people with TB and HCPs in this context. While Viet Nam has begun to implement social support for people with TB, we found that interventions that extend beyond the health system are needed to better protect people with TB against costs and to improve treatment outcomes.

In conjunction with prior research, this study can play an important role in the design of supportive interventions for people with DS- and MDR-TB. Within urban Viet Nam, it can guide how government funds such as PASTB support people with TB with recommendations for actions outlined in Table 4. To expand on this, future research could explore the optimal operational design of interventions, for example by evaluating whether online support groups or psychological training for HCPs are the best way to support people with TB with their mental health.

More broadly, this study emphasises that coordinated action from biomedical, social and economic sectors is needed to support people with TB and other diseases of poverty in Viet Nam and other high burden countries.

Contributorship statement

IS co-developed the research questions, led data analysis (developing the coding framework and identifying themes), drafted and revised the manuscript. RF conceptualised the study, designed topic guides, oversaw data collection, supported and verified analysis, and reviewed the manuscript. She is the guarantor for the paper. KSA conceptualised the study, designed topic guides, co-developed the research questions and supported in analysis. TTN conceptualised the study, oversaw data collection, advised on and reviewed the manuscript. LN, THYP, HN, AJC, LNQV, NN, AK, JC, MP, LB, KL, HBN, NVN edited and reviewed the manuscript. SA co-developed the research questions and the coding framework, supported

For peer review only - http://bmjopen.bmj.com/site/about/guidelines.xhtml

and verified analysis and reviewed the manuscript. All co-authors reviewed and approved the final manuscript.

Acknowledgements

Our special thanks go to the participants in this study for their invaluable contributions to this study. We are particularly grateful to the people with TB for sharing their personal experiences of illness and hope that this study will have a positive impact on people with TB in the future.

We would like to thank the individuals who conducted interviews including: Nham Thi Yen Ngoc, Tran Thi Ngan, and Bui Thi Huyen. Special thanks to the individuals who completed transcriptions, translations and the checking of these processes, including Nguyen Thi Cam Van, Truong Thi Thuy Dung, Nguyen Thi Xuan, Phan Khanh Hang and Huynh Thi Kim Ngan.

We appreciate the staff of the National Lung Hospital, Ha Noi Lung Hospital, and Pham Ngoc Thach Hospital who facilitated interviews for our research team. TB staff based in the following districts of Ha Noi assisted with participant recruitment: Ba Dinh, Dong Da, Hai Ba Trung, Hoan Kiem, and Hoang Mai. In Ho Chi Minh City, we would like to thank staff from five districts who assisted with recruitment including: Binh Chanh, Go Vap, Thu Duc and Districts 6 & 8.

This study was made possible by the generous support of the American people through USAID. The contents are the responsibility of the listed authors, and do not necessarily reflect the views of USAID or the United States Government.

Competing interests: None declared.

Data Availability Statement: Data are available upon reasonable request.

Funding sources: This study was supported by two funders. Interviews, transcriptions and translations among people with DS-TB were funded by the United States Agency for International Development (USAID) Fixed Award No. 72044020FA00001, through the Erase TB Activity. DR-TB interviews were conducted with support from the Stop TB Partnership's TB REACH initiative by Global Affairs Canada grant number CA-3-D000920001 https://w05.international.gc.ca/projectbrowserbanqueprojets/projectprojet/details/d000920001. The funders had no role in study design, data collection and analysis, decision to publish, or preparation of the manuscript.

Ethical considerations: This study was conducted in strict adherence with the Declaration of Helsinki. Ethical approvals were obtained from Ha Noi University School of Public Health (issued on July 17, 2020; IRB #300/2020/YTCC-HD3) and from Pham Ngoc Thach Hospital (issued on September 16, 2020; IRB # 1225/PNT-HĐĐĐ).

For peer review only - http://bmjopen.bmj.com/site/about/guidelines.xhtml

Informed consent was obtained prior to study initiation. At the start of each FGD or KII, a patient information sheet (PIS) and consent form were provided and explained by the moderators. All participants signed the informed consent forms before data collection began and were free to withdraw consent and leave the study at any time.

In terms of infection prevention and control measures, most participants were cured or had completed treatment and participants in the unsuccessful treatment group had been on anti-TB medications for more than one month. Many participants wore masks and hand sanitiser was provided to all participants on entry.

A data-sharing agreement was reached between Friends for International TB Relief (one of the local NGO partners) and the Karolinska Institutet. FIT collected data directly or obtained it through collaboration agreements with local collaborating NGO partners. All data were pseudonymised prior to sharing and stored on a secure, password-protected online platform at Karolinska Institutet.

Figure 1 caption: A conceptual framework for social protection, modified from Sabates-Wheeler and Devereux (30). Thicker lines indicate stronger relationships. The dotted line indicates where measures could have counter-productive effects on social transformation. The top half of the diagram represents policies that can facilitate movement out of poverty (springboards), while the bottom half represents those that alleviate and prevent deprivation (safety nets). Examples relevant to TB in italics.

References

1. World Health Organisation. Global Tuberculosis Report 2022 [Internet]. Geneva; 2022 [cited 2022 Oct 30]. Available from: https://www.who.int/teams/global-tuberculosis-programme/tb-reports/global-tuberculosis-report-2022
2. Lönnroth K, Glaziou P, Weil D, Floyd K, Uplekar M, Raviglione M. Beyond UHC: Monitoring Health and Social Protection Coverage in the Context of Tuberculosis Care and Prevention. PLoS Med [Internet]. 2014 Sep 1 [cited 2022 Jan 30];11(9):e1001693. Available from: https://journals.plos.org/plosmedicine/article?id=10.1371/journal.pmed.1001693
3. Hargreaves JR, Boccia D, Evans CA, Adato M, Petticrew M, Porter JDH. The Social Determinants of Tuberculosis: From Evidence to Action. Am J Public Health [Internet]. 2011 Apr [cited 2022 Apr 30];101(4):654. Available from: /pmc/articles/PMC3052350/
4. Waitt CJ, Squire SB. A systematic review of risk factors for death in adults during and after tuberculosis treatment. International Journal of Tuberculosis and Lung Disease. 2011 Jul;15(7):871–85.
5. Mauch V, Bonsu F, Gyapong M, Awini E, Suarez P, Marcelino B, et al. Free tuberculosis diagnosis and treatment are not enough: patient cost evidence from three continents. Int J Tuberc Lung Dis [Internet]. 2013 Mar 1 [cited 2022 May 2];17(3):381–7. Available from: https://pubmed.ncbi.nlm.nih.gov/23407227/
6. Tanimura T, Jaramillo E, Weil D, Raviglione M, Lönnroth K. Financial burden for tuberculosis patients in low- and middle-income countries: a systematic review. Eur Respir J [Internet]. 2014 [cited 2022 Apr 28];43(6):1763. Available from: /pmc/articles/PMC4040181/
7. Tran PB, Hensing G, Wingfield T, Income security during public health emergencies: the COVID-19 poverty trap in Vietnam, *BMJ Global Health* 2020;5:e002504

For peer review only - http://bmjopen.bmj.com/site/about/guidelines.xhtml

8. World Health Organisation. The end TB strategy [Internet]. Geneva; 2015 [cited 2022 Apr 28]. Available from: https://www.who.int/publications/i/item/WHO-HTM-TB-2015.19
9. Siroka A, Ponce NA, Lönnroth K. The impact of social protection on tuberculosis rates: a global analysis. Lancet Infect Dis [Internet]. 2016 Apr 1 [cited 2022 Apr 28];16(4):473. Available from: /pmc/articles/PMC4834392/
10. Carter DJ, Glaziou P, Lönnroth K, Siroka A, Floyd K, Weil D, et al. The impact of social protection and poverty elimination on global tuberculosis incidence: a statistical modelling analysis of Sustainable Development Goal 1. Lancet Glob Health [Internet]. 2018 May 1 [cited 2022 Jan 30];6(5):e514–22. Available from: https://pubmed.ncbi.nlm.nih.gov/29580761/
11. Boccia D, Hargreaves J, Lönnroth K, Jaramillo E, Weiss J, Uplekar M, et al. Cash transfer and microfinance interventions for tuberculosis control: review of the impact evidence and policy implications. Int J Tuberc Lung Dis [Internet]. 2011 Jun [cited 2022 Apr 28];15 Suppl 2(Suppl 2). Available from: https://pubmed.ncbi.nlm.nih.gov/21740658/
12. Richterman A, Steer-Massaro J, Jarolimova J, Nguyen LBL, Werdenberg J, Ivers LC. Cash interventions to improve clinical outcomes for pulmonary tuberculosis: systematic review and meta-analysis. Bull World Health Organ [Internet]. 2018 Jul 1 [cited 2022 May 4];96(7):471. Available from: /pmc/articles/PMC6022611/
13. de Pee S, Grede N, Mehra D, Bloem MW. The enabling effect of food assistance in improving adherence and/or treatment completion for antiretroviral therapy and tuberculosis treatment: a literature review. AIDS Behav [Internet]. 2014 Oct 1 [cited 2022 May 4];18 Suppl 5:531–41. Available from: https://pubmed.ncbi.nlm.nih.gov/24619602/
14. Wingfield T, Tovar MA, Huff D, Boccia D, Montoya R, Ramos E, et al. A randomized controlled study of socioeconomic support to enhance tuberculosis prevention and treatment, Peru. Bull World Health Organ [Internet]. 2017 Apr 1 [cited 2022 May 4];95(4):270. Available from: /pmc/articles/PMC5407248/
15. Wingfield T, Tovar MA, Huff D, Boccia D, Montoya R, Ramos E, Lewis JJ, Gilman RH, Evans CA. The economic effects of supporting tuberculosis-affected households in Peru. Eur Respir J. 2016 Nov;48(5):1396-1410. doi: 10.1183/13993003.00066-2016. Epub 2016 Sep 22. PMID: 27660507; PMCID: PMC5091496.
16. Todd H, Hudson M, Grolmusova N, Kazibwe J, Pearman J, Skender K, Tran PB, Boccia D, Shete PB, Wingfield T. Social Protection Interventions for TB-Affected Households: A Scoping Review. American Journal of Tropical Medicine and Hygiene [Internet]. 2023. [cited 2023 Apr 4]. Available from: https://doi.org/10.4269/ajtmh.22-0470
17. van Hoorn R, Jaramillo E, Collins D, Gebhard A, van den Hof S. The Effects of Psycho-Emotional and Socio-Economic Support for Tuberculosis Patients on Treatment Adherence and Treatment Outcomes – A Systematic Review and Meta-Analysis. PLoS One [Internet]. 2016 Apr 1 [cited 2022 Apr 28];11(4). Available from: /pmc/articles/PMC4849661/
18. World Health Organization. WHO global lists of high burden countries for TB, multidrug/rifampicin-resistant TB (MDR/RR-TB) and TB/HIV, 2021-20205. Geneva;2021. Available from: https://www.who.int/news/item/17-06-2021-who-releases-new-global-lists-of-high-burden-countries-for-tb-hiv-associated-tb-and-drug-resistant-tb
19. Nguyen HV, Tiemersma EW, Nguyen HB, Cobelens FGJ, Finlay A, Glaziou P, et al. The second national tuberculosis prevalence survey in Vietnam. PLoS One [Internet]. 2020 Apr 1 [cited 2022 Mar 20];15(4):e0232142. Available from: https://journals.plos.org/plosone/article?id=10.1371/journal.pone.0232142

For peer review only - http://bmjopen.bmj.com/site/about/guidelines.xhtml

20. Thi Mai Oanh T, Khanh Phuong N, Anh Tuan K. Sustainability and Resilience in the Vietnamese Health System Sustainability and Resilience in the Vietnamese Health System Sustainability and Resilience in the Vietnamese Health System [Internet]. 2021 [cited 2022 Mar 8]. Available from: https://weforum.org/phssr
21. Phát triển bền vững BHYT toàn dân [Sustainable development of universal health insurance] [Internet]. Bao Hiem Xa Hoi [Vietnam Social Security]. 2019 [cited 2022 Aug 26]. Available from: https://baohiemxahoi.gov.vn/congkhai/Pages/so-lieu-thong-ke-nganh.aspx?CateID=130&ItemID=12916
22. Nhung N v., Hoa NB, Anh NT, Anh LTN, Siroka A, Lönnroth K, et al. Measuring catastrophic costs due to tuberculosis in Viet Nam. International Journal of Tuberculosis and Lung Disease. 2018 Sep 1;22(9):983–90.
23. Tran VT. Circular No. 36/2021/TT-BYT dated December 31, 2021 on Medical examination and treatment, and payment for medical examination and treatment costs covered by health insurance relating to tuberculosis examination and treatment [Internet]. 36/2021/TT-BYT Dec 31, 2021. Available from: https://hethongphapluat.com/circular-no-36-2021-tt-byt-dated-december-31-2021-on-medical-examination-and-treatment-and-payment-for-medical-examination-and-treatment-costs-covered-by-health-insurance-relating-to-tuberculosis-examination-and-treatment.html
24. Hoa NB, Nhung N v. National tuberculosis patients cost survey: research findings lead to change in policy and practice, Viet Nam. Public Health Action [Internet]. 2019 Jul 23 [cited 2022 May 2];9(2):50. Available from: /pmc/articles/PMC6645445/
25. Forse, R., Nguyen, TT., Dam, T., Vo LNQ., Codlin AJ, Viney K., Caws M., Dang HM, Nguyen LH, Nguyen HB, Nguyen NV, Lönnroth K., Sidney Annerstedt, K. A qualitative assessment on the acceptability of providing cash transfers and social health insurance for tuberculosis-affected families in Ho Chi Minh City, Vietnam. Under Review.
26. Tong A, Sainsbury P, Craig J. Consolidated criteria for reporting qualitative research (COREQ): A 32-item checklist for interviews and focus groups. International Journal for Quality in Health Care. 2007 Dec;19(6):349–57.
27. United States Agency for International Development. Fact Sheet: Erase TB Project | Vietnam | U.S. Agency for International Development [Internet]. 2020 [cited 2022 May 11]. Available from: https://www.usaid.gov/vietnam/documents/fact-sheet-erase-tb-project
28. Friends for International TB Relief. Friends for International TB Relief (FIT) [Internet]. [cited 2022 May 11]. Available from: https://tbhilfe.org/
29. Thủ tục hành chính: Đăng ký đóng, cấp thẻ BHYT đối với người chỉ tham gia BHYT [Details of Small Administrative Procedures: Register to pay and issue health insurance cards for people only participating in health insurance] [Internet]. Bao Hiem Xa Hoi [Vietnam Social Security]. [cited 2023 Mar 17]. Available from: https://baohiemxahoi.gov.vn:443/thu-tuc-hanh-chinh/Pages/default.aspx?ItemID=73
30. Vuong Q-H, La V-P, Nguyen M-H, Nguyen T-HT, Ho M-T. Good budget or good care: The dilemma of social health insurance in Vietnam. *SAGE Open Medicine*. 2021;9. doi:10.1177/20503121211042512
31. Malterud K, Siersma VD, Guassora AD. Sample Size in Qualitative Interview Studies: Guided by Information Power. Qual Health Res [Internet]. 2016 Nov 1 [cited 2022 Mar 8];26(13):1753–60. Available from: https://journals-sagepub-com.proxy.kib.ki.se/doi/full/10.1177/1049732315617444

For peer review only - http://bmjopen.bmj.com/site/about/guidelines.xhtml

32. Braun V, Clarke V. Using thematic analysis in psychology. Qual Res Psychol [Internet]. 2006 [cited 2022 Jan 30];3(2):77–101. Available from: https://www.tandfonline.com/action/journalInformation?journalCode=uqrp20
33. Braun V, Clarke V. Reflecting on reflexive thematic analysis. Qual Res Sport Exerc Health [Internet]. 2019;11(4):589–97. Available from: https://www.tandfonline.com/action/journalInformation?journalCode=rqrs21
34. Dedoose version 9.0.17, web application for managing, analysing and presenting qualitative and mixed methods research data. Los Angeles, CA: SocioCultural Research Consultants, LLC; 2021.
35. Sabates-Wheeler R, Devereux S. Social protection for transformation. IDS Bull. 2007 May;38(3):23–8.
36. Forse R, Yoshino CA, Nguyen TT, Phan THY, Vo LNQ, Codlin AJ, Nguyen L, Hoang C, Basu L, Pham M, Nguyen HB, Dinh LV, Caws M, Viney K, Wingfield T, Lonnroth K, Sidney-Annerstedt K. Towards Universal Health Coverage in Viet Nam: A mixed-method case study of enrolling people with tuberculosis into social health insurance.Under Review.
37. Boccia D, Pedrazzoli D, Wingfield T, Jaramillo E, Lönnroth K, Lewis J, et al. Towards cash transfer interventions for tuberculosis prevention, care and control: key operational challenges and research priorities. BMC Infect Dis [Internet]. 2016 Jun 21 [cited 2022 Apr 28];16(1). Available from: /pmc/articles/PMC4915091/
38. Gentilini U. The Revival of the ‘Cash versus Food’ Debate New Evidence for an Old Quandary? [Internet]. 2016 [cited 2022 May 4]. Available from: http://econ.worldbank.org.
39. United States Agency for International Development. Fact Sheet: Erase TB Project | Vietnam | U.S. Agency for International Development [Internet]. 2020 [cited 2022 May 11]. Available from: https://www.usaid.gov/vietnam/documents/fact-sheet-erase-tb-project
40. Ciobanu A, Domente L, Soltan V, Bivol S, Severin L, Plesca V, et al. Do incentives improve tuberculosis treatment outcomes in the Republic of Moldova? Public Health Action [Internet]. 2014 Oct 21 [cited 2022 Sep 19];4(Suppl 2):S59–63. Available from: https://pubmed.ncbi.nlm.nih.gov/26393100/
41. Lu H, Yan F, Wang W, Wu L, Ma W, Chen J, et al. Do transportation subsidies and living allowances improve tuberculosis control outcomes among internal migrants in urban Shanghai, China? Western Pac Surveill Response J [Internet]. 2013 Jan 1 [cited 2022 May 10];4(1):19–24. Available from: https://pubmed.ncbi.nlm.nih.gov/23908951/
42. Vanleeuw L, Zembe-Mkabile W, Atkins S. “I’m suffering for food”: Food insecurity and access to social protection for TB patients and their households in Cape Town, South Africa. Vousden G, editor. PLoS One [Internet]. 2022 Apr 26 [cited 2022 May 2];17(4):e0266356. Available from: /pmc/articles/PMC9041827/
43. Grobler L, Nagpal S, Sudarsanam TD, Sinclair D. Nutritional supplements for people being treated for active tuberculosis. Cochrane Database Syst Rev. 2016 Jun 29;2016(6):CD006086. doi: 10.1002/14651858.CD006086.pub4. PMID: 27355911; PMCID: PMC4981643.
44. Liu Q, Garner P, Wang Y, Huang B, Smith H. Drugs and herbs given to prevent hepatotoxicity of tuberculosis therapy: systematic review of ingredients and evaluation studies. BMC Public Health. 2008 Oct 21;8:365. doi: 10.1186/1471-2458-8-365. PMID: 18939987; PMCID: PMC2576232.
45. Meghji J, Gregorius S, Madan J, Chitimbe F, Thomson R, Rylance J, et al. The long term effect of pulmonary tuberculosis on income and employment in a low income, urban setting. Thorax [Internet]. 2021 Apr 1 [cited 2022 May 4];76(4):387–95. Available from: https://pubmed.ncbi.nlm.nih.gov/33443228/

For peer review only - http://bmjopen.bmj.com/site/about/guidelines.xhtml

46. General Statistics Office of Viet Nam. Report on the Covid-19 impacts on labour and employment situation in the third quarter of 2021 [Internet]. Ha Noi; 2021 Oct [cited 2022 Sep 13]. Available from: https://www.gso.gov.vn/en/data-and-statistics/2021/10/report-on-the-covid-19-impacts-on-labour-and-employment-situation-in-the-third-quarter-of-2021/
47. Thomas BE, Shanmugam P, Malaisamy M, Ovung S, Suresh C, Subbaraman R, et al. Psycho-Socio-Economic Issues Challenging Multidrug Resistant Tuberculosis Patients: A Systematic Review. PLoS One [Internet]. 2016 Jan 1 [cited 2022 May 3];11(1):e0147397. Available from: https://journals.plos.org/plosone/article?id=10.1371/journal.pone.0147397
48. Long NH, Johansson E, Diwan VK, Winkvist A. Fear and social isolation as consequences of tuberculosis in VietNam: a gender analysis. Health Policy (New York). 2001 Oct 1;58(1):69–81.
49. Chalco K, Wu DY, Mestanza L, Muñoz M, Llaro K, Guerra D, et al. Nurses as providers of emotional support to patients with MDR-TB. Int Nurs Rev [Internet]. 2006 Dec [cited 2022 May 5];53(4):253–60. Available from: https://pubmed.ncbi.nlm.nih.gov/17083413/
50. Baral SC, Aryal Y, Bhattrai R, King R, Newell JN. The importance of providing counselling and financial support to patients receiving treatment for multi-drug resistant TB: mixed method qualitative and pilot intervention studies. BMC Public Health [Internet]. 2014 Jan 17 [cited 2022 May 5];14(1):46. Available from: /pmc/articles/PMC3898066/
51. Vo LNQ, Forse RJ, Codlin A, Dang HA, Truong V v., Nguyen LJ, et al. Socio-protective effects of active case finding on catastrophic costs from tuberculosis in Ho Chi Minh City, Viet Nam: a longitudinal patient cost survey. BMC Health Serv Res [Internet]. 2021 [cited 2022 Oct 24];21(1051). Available from: https://doi.org/10.1186/s12913-021-06984-2
52. Roy M, Bolton Moore C, Sikazwe I, Holmes CB. A Review of Differentiated Service Delivery for HIV Treatment: Effectiveness, Mechanisms, Targeting, and Scale. Vol. 16, Current HIV/AIDS Reports. 2019.
53. Hollander JA. The Social Contexts of Focus Groups. SAGE. 2016 Jul 33(5):602–37.
54. Moynihan R, Sanders S, Michaleff ZA, Scott AM, Clark J, To EJ, et al. Impact of COVID-19 pandemic on utilisation of healthcare services: a systematic review. BMJ Open [Internet]. 2021 Mar 1 [cited 2022 May 5];11(3):e045343. Available from: https://bmjopen.bmj.com/content/11/3/e045343
55. Tran BX, Nguyen HT, Le HT, Latkin CA, Pham HQ, Vu LG, et al. Impact of COVID-19 on Economic Well-Being and Quality of Life of the Vietnamese During the National Social Distancing. Frontiers in Psychology. 2020 Sep 11;11:2289.
56. Smith HJ, Chen J, Liu X. Language and rigour in qualitative research: Problems and principles in analyzing data collected in Mandarin. BMC Medical Research Methodology [Internet]. 2008 Jul 10 [cited 2022 May 4];8(1):1–8. Available from: https://bmcmedresmethodol.biomedcentral.com/articles/10.1186/1471-2288-8-44

For peer review only

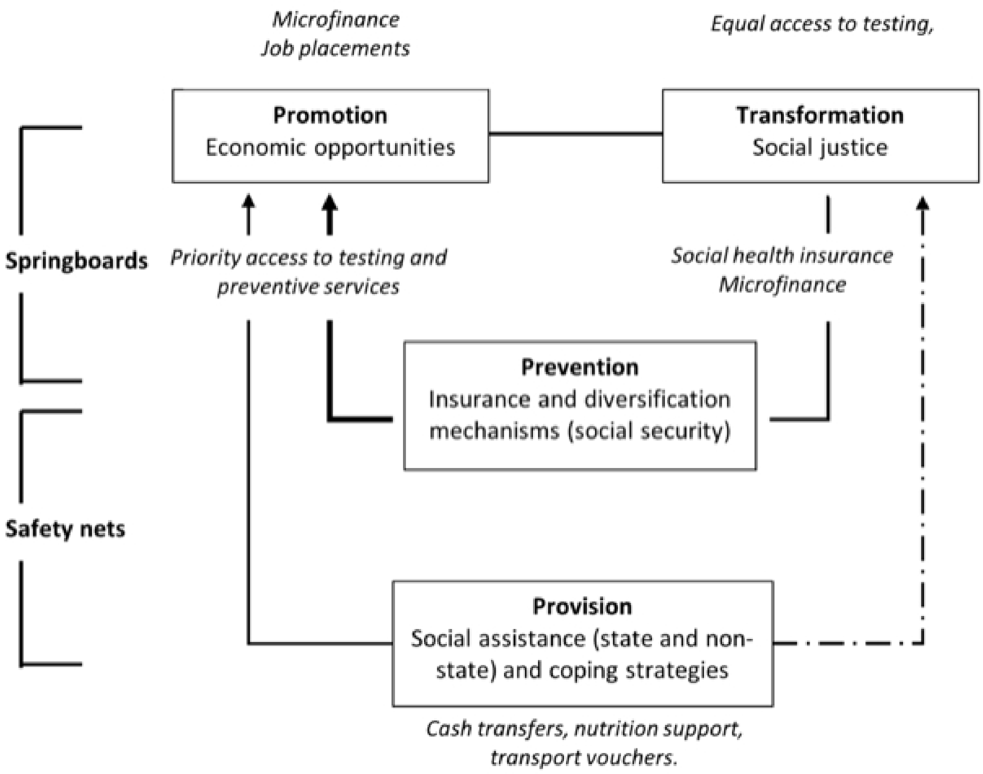

Figure 1: A conceptual framework for social protection, adapted from Sabates-Wheeler and Devereux (30). Thicker lines indicate stronger relationships. The dotted line indicates where measures could have counter-productive effects on social transformation. The top half of the diagram represents policies that can facilitate movement out of poverty (springboards), while the bottom half represents those that alleviate and prevent deprivation (safety nets). Examples relevant to TB in italics.

108x84mm (144 x 144 DPI)

Appendix A: Details of data collection

**Participant group**	**Number of participants**	**Type**	**Location**	**Duration** (minutes)
People with successfully treated DS-TB	5¶	FGD	Ha Noi (NGO office)	146
People with unsuccessfully treated DS-TB	4	FGD	Ha Noi (NGO office)	103
District TB Unit HCP	5	FGD	Ha Noi (NGO office)	91
People with successfully treated DS-TB	3	FGD	Ho Chi Minh City (NGO office)	138
People with unsuccessfully treated DS-TB	2	FGD	Ho Chi Minh City (NGO office)	113
People with MDR-TB	4	FGD	Ha Noi (Ha Noi Lung Hospital)	109
People with MDR-TB	6	FGD	Ho Chi Minh City (NGO office)	188
National/Provincial TB HCP	4*	KII	Ha Noi (Zoom)	46-90

¶ Indicates that at least one participant was accompanied by their caregiver
**Four separate KII were conducted.*

For peer review only

Appendix B: Topic guides for data collection

Focus Group Discussions (DTU and Provincial TB hospital staff)

Prior experiences of providing TB treatment support

- What existing social support resources are available for the TB patients that you treat?
    - How do you link your patients to existing resources?

Barriers to TB treatment

- From your experience, what is the biggest obstacle that prevents patients from completing treatment?
- What are the main factors that cause catastrophic costs or poverty during TB treatment?

Types of social support

- If you could design any intervention to support the TB patients you treat, what would be provided?

Social health insurance

- Which types of TB patients should receive health insurance? Why?
- Who should provide SHI to TB patients?
- How effective is SHI at alleviating the costs of TB care?

TB Education

- On what types of information should TB patients receive counselling and education?
- When should this information be given during the duration of treatment?
- If you were given any teaching aids to improve the patient counselling, what would those teaching aids look like?

Nutritional support

- What types of food are needed by TB patients?
- What types of supplements are needed during TB treatment?
- Which types of patients should be offered nutritional support, if it is offered?
- For how long do TB patients need nutritional support?
    - Prompt: during the intensive phase or throughout all of treatment?
- What are the benefits of providing food and/or supplements?
- Do you see any drawbacks to the provision of food and/or supplements?

For peer review only

Cash transfers

- Which types of TB patients should receive the cash transfer? Why?
- For how long should cash be given to TB patients?
- What are the benefits and drawbacks of cash transfers compared to other types of social support?
- How should cash be spent?
- How should cash not be spent?

Loans

- What was your need for credit during TB care?
- How do you feel about providing loans to TB patients? Whose role should it be to provide loans?
- How much credit should be given?
- How should TB patients spend the loan money?
- How would loans address the needs of TB patients?
- What should the payment plan consist of?
- How should we collect on the loan?

Transport vouchers

- How do you feel about transportation vouchers being given to TB patients?
- How do transport vouchers address the needs of TB patients?
- Which types of patients should be offered transport vouchers, if they are offered?
- How willing do you feel TB patients would be to participate in a project that provided transport vouchers?
- What challenges do you foresee in providing transport vouchers to TB patients?

Patient support groups

- What is your opinion of TB patient support groups?
- Which types of TB patients would benefit the most from patient support groups?
- What types of benefits would TB patients gain from a TB support group?
- How willing do you feel TB patients would be to participate patient support groups?
- During which stage of TB treatment would a patient support group be most effective to support the patient?
- Who is the most effective type of person to lead the patient support group?

For peer review only

- Prompt: Peer-led, DTU-led or psychologist-led
- How would you feel about hosting a patient support group?

Access to a psychologist/ psychiatrist for DS-TB patients

- What types of psychological or psychiatric issues do your DS-TB patients face?
- What mental health support services do DS-TB patients require?
- What resources are currently available to them for psychological or psychiatric problems?
- How willing do you feel DS-TB patients would be to meet with a mental health professional during their treatment?
- How would better access to a mental health professional improve DS-TB treatment outcomes?

Priority access to testing and preventative therapy

- How can patient's families best be supported to go for TB testing?
- What barriers do families of people with TB face in accessing TB testing?
- What is the best way to improve LTBI treatment initiation rates among TB contacts?
- What barriers do families of people with TB face in accessing LTBI testing and treatment?

Prioritization to improve treatment outcomes

- Following this discussion, what do you feel is the most important type of support for TB patients to improve their treatment outcomes? Why?
- What are the second and third most important types of support for TB treatment?

Prioritization to reduce catastrophic costs

- What do you feel is the most important type of support for TB patients to reduce catastrophic costs? Why?
- What are the second and third most important types of support to reduce patient costs?

Is there anything else you would like to tell us?

Focus Group Discussions (Current/Former TB patients)

Experiences on TB treatment

- When you were on TB treatment, what types of challenges did you face?
- What types of support did you need? Why?

For peer review only

Barriers to TB treatment

- From your experience, what is the biggest obstacle that prevents patients from completing treatment?
- What are the main factors that cause catastrophic costs or poverty during TB treatment?

Types of support

- If you could design any type of program to help TB patients, not thinking about the cost, what would that program include?

Social health insurance

- Which types of TB patients should receive health insurance? Why?
- Who should provide SHI to TB patients?
- How effective is SHI at alleviating the costs of TB care?

Education

- On what types of information should TB patients receive counselling?
- When should this information be given during the duration of treatment?
- Thinking back the to the counselling you received, if you could change anything about this counselling, what would you change?

Nutritional support

- What types of food are needed by TB patients?
- What types of supplements are needed during TB treatment?
- Which types of patients should be offered nutritional support, if it is offered?
- For how long do TB patients need nutritional support?
    - Prompt: during the intensive phase or throughout all of treatment?
- What are the benefits of providing food and/or supplements?
- Do you see any drawbacks to the provision of food and/or supplements?

Cash transfers

- Which types of TB patients should receive the cash transfer? Why?
- For how long should cash be given to TB patients?
- What are the benefits and drawbacks of cash transfers compared to other types of social support?

For peer review only

- How should cash be spent?
- How should cash not be spent?

Vocational training

- How helpful do you feel vocational training would be to TB patients?
  - Prompt: During treatment vs. after treatment
  - Prompt: short-term vs. long-term benefits
- Which types of patients should be offered vocational training, if it is offered?
- How willing do you feel TB patients would be to participate?
- What would be the benefits of vocational training to TB patients?
- Which barriers would TB patients face completing the vocational training?

Transport vouchers

- How do you feel about transportation vouchers being given to TB patients?
- How do transport vouchers address the needs of TB patients?
- Which types of patients should be offered transport vouchers, if they are offered?
- How willing do you feel TB patients would be to participate in a project that provided transport vouchers?
- What challenges do you foresee in providing transport vouchers to TB patients?
- What are the benefits of transport vouchers compared to cash payments?

Patient support groups

- What is your opinion of TB patient support groups?
- What types of challenges do patient support groups best address?
- What issues should patient support groups discuss?
- Which types of TB patients would benefit the most from patient support groups?
- What types of benefits would TB patients gain from a TB support group?
- How willing do you feel TB patients would be to participate patient support groups?
- During which stage of TB treatment would a patient support group be most effective to support the patient?
- Who is the most effective type of person to lead the patient support group?
  - Prompt: Peer-led, DTU-led or psychologist-led
- How would you feel about hosting a patient support group?

Access to a psychologist/ psychiatrist for DS-TB patients

For peer review only

- What mental health support services did you require during your treatment?
- What resources were available to you for psychological or psychiatric problems, if any?
- How willing do you feel DS-TB patients would be to meet with a mental health professional during their treatment?
- How would access to a mental health professional improve DS-TB treatment outcomes?

Access to testing and preventative therapy

- How can patient's families best be supported to go for TB testing?
- What barriers do families of people with TB face in accessing TB testing?
- What is the best way to improve LTBI treatment initiation rates among TB contacts?
- What barriers do families of people with TB face in accessing LTBI testing and treatment?

Prioritization to improve treatment outcomes

- Following this discussion, what do you feel is the most important type of support for TB patients to improve their treatment outcomes? Why?
- What are the second and third most important types of support for TB treatment?

Prioritization to reduce catastrophic costs

- What do you feel is the most important type of support for TB patients to reduce catastrophic costs? Why?
- What are the second and third most important types of support to reduce patient costs?

Is there anything else you would like to tell us?

For peer review only

KIIs (National and Provincial TB staff)

Barriers

- From your experience, what is the biggest obstacle that prevents patients from completing treatment?
- What are the main factors that cause catastrophic costs or poverty during DR-TB treatment?

Prior experiences of providing treatment support to people with DR-TB

- What existing support resources are available for the DR-TB patients that you treat?
    - How do you link your patients to existing resources?
    - How effective do you find these sources to be?
        - Describe the gaps in this support
        - How adequate do you find this support to be?
        - How easy or difficult is it for people with DR-TB to obtain economic or psychological support?
- Which groups of people with DR-TB require economic and psychological support?

Intervention design

- If you could design any intervention to support DR-TB patients, what would be provided?
    - Why would this be provided?
    - Who should receive it?
        - Differentiate packages of support based on levels of disability/ poverty status/ income/ number of prior episodes of TB?
    - For how long should this support be provided?
    - What should be required of the DR-TB patient to receive the support?
    - What should *not* be provided?
    - How can technology be used to support this intervention?
        - Prompt for remote monitoring and drug delivery through shipper apps

Priortization to improve treatment outcomes & catastrophic costs

- Even if this differs from the intervention you proposed, what do you feel is the most important type of support for DR-TB patients to improve DR-TB *treatment outcomes*? Why?
- What do you feel is the most important type of support for DR-TB patients to reduce *catastrophic costs*? Why?

For peer review only

Provision of support

- In your role, do you have capacity to assist with the provision of support to people with DR-TB?
- If this work was to be conducted in the facility where you work, who should provide it?

Is there anything else you would like to tell us?

For peer review only

Focus Group Discussions (Current/Former DR-TB patients)

Experiences on TB treatment

- While on treatment, what types of challenges have you faced?
- What types of support do you need during DR-TB treatment? Why?

Barriers to TB treatment

- From your experience, what is the biggest obstacle that prevents patients from completing treatment?
- What are the main factors that cause catastrophic costs or poverty during DR-TB treatment?

Types of support

- If you could design any type of program to help people through DR-TB treatment, not thinking about the cost, what would that program include?

***If the participants mention a type of support listed below, then ask the questions for that form of support. If they do not bring up a form of support that is outlined below, then prompt them to discuss.*

Social health insurance

- Which types of DR-TB patients should receive health insurance? Why?
- Who should provide SHI to DR-TB patients?
- For how long should SHI be provided to people with DR-TB?
- How effective is SHI at alleviating the costs of DR-TB care?

Education

- On what types of information should DR-TB patients receive counselling?
- When should this information be given during the duration of treatment?
- Thinking back the to the counselling you received, if you could change anything about this counselling, what would you change?

Nutritional support

- What types of food are needed for people with DR-TB?
- What types of supplements are needed during DR-TB treatment?
- Which types of people with DR-TB should be offered nutritional support, if it is offered?

For peer review only

   - Differentiate packages of support based on levels of disability/ poverty status/ income/ number of prior episodes of TB?
- For how long do DR-TB patients need nutritional support?
   - Prompt: during the intensive phase or throughout all of treatment?
- What are the benefits of providing food and/or supplements?
- Do you see any drawbacks to the provision of food and/or supplements?

<u>Cash transfers</u>

- Which types of DR-TB patients should receive the cash transfer? Why?
   - Differentiate cash amounts based on levels of disability/ poverty status/ income/ number of prior episodes of TB?
- For how long should cash be given to people with DR-TB?
- What are the benefits and drawbacks of cash transfers compared to other types of social support?
- How should cash be spent?
- How should cash not be spent?

<u>Transport vouchers</u>

- How do you feel about transportation vouchers being given to people with DR-TB?
- How do transport vouchers address the needs of people with DR-TB?
- Which types of patients should be offered transport vouchers, if they are offered?
   - Differentiate amount of transport voucher based on distance from clinic/ levels of disability/ poverty status/ income/ number of prior episodes of TB?
- How willing do you feel DR-TB patients would be to participate in a project that provided transport vouchers?
- For how long should transport vouchers be given to people with DR-TB?
- What are the benefits of transport vouchers compared to cash payments?

<u>At-home medicine delivery</u>

- How do you feel about medicine being delivered to the homes of people with DR-TB through an app such as Grab, Ahamove, Bee, or another shipper?
- How would medicine delivery address the needs of people with DR-TB?
- Which types of patients should be offered medicine delivery?
- How willing do you feel DR-TB patients would be to receive medicine delivery?
- For how long should medicine be delivered to the homes of people with DR-TB?

For peer review only

Remote monitoring

- How do you feel about attending appointments with your doctor online?
- How would online appointments address the needs of people with DR-TB?
- Which types of patients should be offered remote monitoring or telemedicine?
- How willing do you feel DR-TB patients would be to receive online consultations of in-person meetings?

Patient support groups

- What is your opinion of DR-TB patient support groups?
    - Prompt- in person vs. online support groups
- What types of challenges do patient support groups best address?
- What issues should patient support groups discuss?
- Which types of DR-TB patients would benefit the most from patient support groups?
- What types of benefits would DR-TB patients gain from a support group?
- How willing do you feel people with DR-TB would be to participate patient support groups?
    - Prompt- in person vs. online support groups
- During which stage of DR-TB treatment would a patient support group be most effective to support the patient?
- Who is the most effective type of person to lead the patient support group?
    - Prompt: Peer-led, DTU-led or psychologist-led
- How would you feel about hosting a patient support group?

Access to a psychologist/ psychiatrist for DR-TB patients

- What mental health support services have you required during your treatment?
- What resources were available to you for psychological or psychiatric problems, if any?
- How willing do you feel DR-TB patients would be to meet with a mental health professional during their treatment?
- How would access to a mental health professional improve DR-TB treatment outcomes?

Access to testing and preventative therapy

- How can patient's families best be supported to go for TB testing?
- What barriers do families of people with TB face in accessing TB testing?
- What is the best way to improve LTBI treatment initiation rates among DR-TB contacts?

For peer review only

- What barriers do families of people with DR-TB face in accessing LTBI testing and treatment?

Priortization to improve treatment outcomes & catastrophic costs

- Following this discussion, what do you feel is the most important type of support for DR-TB patients to improve their *treatment outcomes*? Why?
- What do you feel is the most important type of support for TB patients to reduce catastrophic costs? Why?

Is there anything else you would like to tell us?

For peer review only

COREQ (COnsolidated criteria for REporting Qualitative research) Checklist

A checklist of items that should be included in reports of qualitative research. You must report the page number in your manuscript where you consider each of the items listed in this checklist. If you have not included this information, either revise your manuscript accordingly before submitting or note N/A.

Topic	Item No.	Guide Questions/Description	Reported on Page No.
**Domain 1: Research team and reflexivity**			
Personal characteristics			
Interviewer/facilitator	1	Which author/s conducted the interview or focus group?	5
Credentials	2	What were the researcher's credentials? E.g. PhD, MD	5
Occupation	3	What was their occupation at the time of the study?	5
Gender	4	Was the researcher male or female?	5
Experience and training	5	What experience or training did the researcher have?	5
Relationship with participants			
Relationship established	6	Was a relationship established prior to study commencement?	4
Participant knowledge of the interviewer	7	What did the participants know about the researcher? e.g. personal goals, reasons for doing the research	4
Interviewer characteristics	8	What characteristics were reported about the inter viewer/facilitator? e.g. Bias, assumptions, reasons and interests in the research topic	5
**Domain 2: Study design**			
Theoretical framework			
Methodological orientation and Theory	9	What methodological orientation was stated to underpin the study? e.g. grounded theory, discourse analysis, ethnography, phenomenology, content analysis	5
Participant selection			
Sampling	10	How were participants selected? e.g. purposive, convenience, consecutive, snowball	4
Method of approach	11	How were participants approached? e.g. face-to-face, telephone, mail, email	4
Sample size	12	How many participants were in the study?	4
Non-participation	13	How many people refused to participate or dropped out? Reasons?	4
Setting			
Setting of data collection	14	Where was the data collected? e.g. home, clinic, workplace	5
Presence of non-participants	15	Was anyone else present besides the participants and researchers?	4
Description of sample	16	What are the important characteristics of the sample? e.g. demographic data, date	4-5
Data collection			
Interview guide	17	Were questions, prompts, guides provided by the authors? Was it pilot tested?	5
Repeat interviews	18	Were repeat inter views carried out? If yes, how many?	5
Audio/visual recording	19	Did the research use audio or visual recording to collect the data?	5
Field notes	20	Were field notes made during and/or after the inter view or focus group?	5
Duration	21	What was the duration of the inter views or focus group?	5
Data saturation	22	Was data saturation discussed?	4
Transcripts returned	23	Were transcripts returned to participants for comment and/or	5

For peer review only

Topic	Item No.	Guide Questions/Description	Reported on Page No.
		correction?	
**Domain 3: analysis and findings**			
Data analysis			
Number of data coders	24	How many data coders coded the data?	5
Description of the coding tree	25	Did authors provide a description of the coding tree?	5
Derivation of themes	26	Were themes identified in advance or derived from the data?	5
Software	27	What software, if applicable, was used to manage the data?	5
Participant checking	28	Did participants provide feedback on the findings?	5
Reporting			
Quotations presented	29	Were participant quotations presented to illustrate the themes/findings? Was each quotation identified? e.g. participant number	7-10
Data and findings consistent	30	Was there consistency between the data presented and the findings?	7-10
Clarity of major themes	31	Were major themes clearly presented in the findings?	7-10
Clarity of minor themes	32	Is there a description of diverse cases or discussion of minor themes?	7-10

Developed from: Tong A, Sainsbury P, Craig J. Consolidated criteria for reporting qualitative research (COREQ): a 32-item checklist for interviews and focus groups. *International Journal for Quality in Health Care*. 2007. Volume 19, Number 6: pp. 349 – 357

Once you have completed this checklist, please save a copy and upload it as part of your submission. DO NOT include this checklist as part of the main manuscript document. It must be uploaded as a separate file.

For peer review only
